# Rapid prefrontal cortex activation towards aversively paired faces and enhanced contingency detection are observed in highly trait-anxious women under challenging conditions

**DOI:** 10.3389/fnbeh.2015.00155

**Published:** 2015-06-10

**Authors:** Maimu Alissa Rehbein, Ida Wessing, Pienie Zwitserlood, Christian Steinberg, Annuschka Salima Eden, Christian Dobel, Markus Junghöfer

**Affiliations:** ^1^Institute for Biomagnetism and Biosignalanalysis, University Hospital MünsterMünster, Germany; ^2^Otto Creutzfeldt Center for Cognitive and Behavioral Neuroscience, University of MünsterMünster, Germany; ^3^Department of Child and Adolescent Psychiatry, University Hospital MünsterMünster, Germany; ^4^Institute of Psychology, University of MünsterMünster, Germany

**Keywords:** trait anxiety, contingency awareness, working memory, classical conditioning, magnetoencephalography, MEG, EEG

## Abstract

Relative to healthy controls, anxiety-disorder patients show anomalies in classical conditioning that may either result from, or provide a risk factor for, clinically relevant anxiety. Here, we investigated whether healthy participants with enhanced anxiety vulnerability show abnormalities in a challenging affective-conditioning paradigm, in which many stimulus-reinforcer associations had to be acquired with only few learning trials. Forty-seven high and low trait-anxious females underwent MultiCS conditioning, in which 52 different neutral faces (CS+) were paired with an aversive noise (US), while further 52 faces (CS−) remained unpaired. Emotional learning was assessed by evaluative (rating), behavioral (dot-probe, contingency report), and neurophysiological (magnetoencephalography) measures before, during, and after learning. High and low trait-anxious groups did not differ in evaluative ratings or response priming before or after conditioning. High trait-anxious women, however, were better than low trait-anxious women at reporting CS+/US contingencies after conditioning, and showed an enhanced prefrontal cortex (PFC) activation towards CS+ in the M1 (i.e., 80–117 ms) and M170 time intervals (i.e., 140–160 ms) during acquisition. These effects in MultiCS conditioning observed in individuals with elevated trait anxiety are consistent with theories of enhanced conditionability in anxiety vulnerability. Furthermore, they point towards increased threat monitoring and detection in highly trait-anxious females, possibly mediated by alterations in visual working memory.

## Introduction

Classical conditioning describes a learning process in which a neutral conditioned stimulus (CS) adopts the emotional value of an unconditioned stimulus (US) after being reliably paired with the US (Pavlov, 1927). In the etiology of anxiety disorders, classical conditioning is considered a potential mechanism, although it remains unclear to what degree it specifically contributes to the development of anxiety disorders (Pape and Pare, 2010). Nevertheless, compared to healthy controls, anxiety-disorder patients show anomalies in this type of learning (see Lissek et al., 2005, for a meta-analysis), which could either reflect abnormalities that enhance the risk of developing an anxiety disorder, or be a result of the disease itself. To study whether aberrations in classical conditioning contribute to anxiety vulnerability, individuals at risk to develop, but not yet suffering from an anxiety disorder, should be investigated. Certain personality traits, such as neuroticism or trait anxiety, may increase anxiety vulnerability (Gershuny and Sher, 1998; Jorm et al., 2000; McNally et al., 2003). Persons with high relative to low trait anxiety interpret more situations as threatening and react with increased state anxiety (Laux et al., 1981).

Among the different explanations for a heightened anxiety risk in highly trait-anxious individuals (see Lissek et al., 2005, for an overview) the most prominent are *enhanced conditionability* (e.g., Pitman and Orr, 1986; Orr et al., 2000; Peri et al., 2000; Otto et al., 2007) and *insufficient inhibitory learning* (e.g., Davis et al., 2000; Hermann et al., 2002). Theories of enhanced conditionability assume that CS+/US associations are more easily learned, and/or more robust against extinction, in anxiety-vulnerable relative to invulnerable subjects. Indeed, a recent study reported that high trait anxiety increased the probability of conditioned eye-blink responses during fear acquisition (Caulfield et al., 2013). Theories of impaired safety learning propose that anxiety-vulnerable individuals are less effective than invulnerable ones in inhibiting fear responses to stimuli that do not signal danger. For example, highly trait-anxious participants revealed enhanced self-reported distress and startle responses compared to low-trait anxious participants to a stimulus not paired with the US during conditioning (Gazendam et al., 2013). Note that some studies found no effects of trait anxiety on classical conditioning (Joos et al., 2012; Arnaudova et al., 2013; Torrents-Rodas et al., 2013). Beckers et al. (2013) as well as Gazendam et al. (2013) proposed that such divergent results could be due to the use of different study designs and response measures. Furthermore, they suggested that effects of trait anxiety on classical conditioning should be assessed with several response measures, since fear induces changes on various dimensions. Moreover, it has been argued that ambiguous testing situations, in which the contingencies between the CSs and US are not well established, are more useful in revealing individual differences in classical conditioning (Lissek et al., 2006; Arnaudova et al., 2013; Beckers et al., 2013; Gazendam et al., 2013).

Here, we used such an ambiguous learning situation, *MultiCS conditioning*, to investigate the effect of high vs. low trait anxiety on conditioned responding during fear acquisition and extinction. In MultiCS conditioning, many neutral stimuli (CS+) are paired with an affective US, while the same number of stimuli (CS−) remain unpaired or are paired with a neutral US. This procedure ensures many different stimuli per affective category (CS+, CS−), so that each affective category can be presented multiple times without having to repeat individual stimuli very often (see Steinberg et al., 2013, for more information on this paradigm). Since acquisition of multiple stimulus-reinforcer associations relies on only few CS+/US pairings, awareness of contingencies is strongly reduced (Bröckelmann et al., 2011, 2013; Steinberg et al., 2012, 2013; Rehbein et al., 2014). Nevertheless, CS+ are rated as less pleasant than CS− after conditioning (Klinkenberg et al., 2011; Steinberg et al., 2012; Rehbein et al., 2014), and changes in CS valence facilitate reactions in a forced-choice valence decision task (Bröckelmann et al., 2013). Magnetoencephalography (MEG), comparing measurements after and before MultiCS conditioning, reveals that CS+ evoke more activation of prefrontal cortex (PFC) regions than CS−, at very early processing stages (<100 ms; e.g., Steinberg et al., 2013). Rehbein et al. (2014) showed that this enhanced CS+ activation in PFC regions emerges rapidly, that is, after a single learning trial. Thus, MultiCS conditioning provides an ambiguous learning paradigm that reveals characteristic changes in short-latency neuronal processing, evaluative ratings, and behavioral orienting towards CS+ vs. CS−, despite participants’ inability to report contingencies.

The typical combination of many CSs and few learning trials in MultiCS conditioning challenges the human capacity of contingency learning. Against the classic separation of cognition and emotion, there is accumulating evidence for interactions between the two factors, concerning their influence on neuronal information-processing and their reliance on executive functions (see Berggren and Derakshan, 2013, for a review). For example, the dual competition model (cf., Pessoa, 2010) proposes that the execution of cognitive processes and the prioritization of highly salient affective stimuli draw on shared resources, both engaging similar neuronal structures, such as the frontoparietal attention network. Thus, the processing of highly salient affective stimuli may interrupt ongoing cognitive processes, but challenging cognitive tasks may also impede processing of emotional stimuli. These are important considerations, also in the context of classical conditioning. Cosand et al. (2008) and Baas (2013) showed that awareness of CS+/US associations relies partly on working-memory capacity and attentional control. In an elegant study, Raes et al. (2009) revealed reduced extinction of CS+/US associations under high vs. low cognitive load, but high cognitive load interrupted extinction more in low- than high-anxious participants. They argued that high- relative to low-anxious participants attributed more attentional resources to the detection of threatening stimuli, which facilitated the learning of an inhibitory CS+/noUS association during extinction. Moreover, Moriya and Sugiura (2012) found that visual working-memory capacity is overall increased in high-trait (socially) anxious subjects, but their performance is easily disturbed by task-irrelevant distractors.

In this study, we evaluated differences between high and low trait-anxious participants in emotional learning, using an ambiguous and cognitively challenging MultiCS conditioning procedure, with multiple neutral faces serving as CSs, and an aversive noise as US. Neuronal processing of CS faces was recorded with MEG during habituation, fear acquisition, and extinction. Subjective ratings of valence and arousal and a dot-probe task assessed evaluative changes and behavioral orienting towards CS+ after learning. Participants also indicated their contingency awareness in a matching task. Due to the use of multiple response measures and the ambiguity of the paradigm, we expected to observe differences in fear conditioning between the high and low trait-anxious subjects. If high trait anxiety is associated with enhanced conditionability, we expected the high trait-anxious group to show a more pronounced enhancement of early PFC activation towards CS+ vs. CS− than the low-anxious group, during fear acquisition and extinction. Likewise, evaluative changes in subjective ratings and behavioral orienting towards CS+ in the dot-probe task should be much larger in high relative to low trait-anxious participants. In contrast, if high trait anxiety is associated with reduced fear inhibition, the high trait-anxious group should show less differentiation of CS+ and CS− stimuli than the low-anxious group. In this case, the enhanced activation of CS+ vs. CS− in the PFC during early time windows, the change in evaluative ratings, and the degree of behavioral orienting towards CS+ should be more pronounced in the low than in the high trait-anxiety group. Given the high cognitive challenge of the paradigm, and previous data from MultiCS conditioning (e.g., Steinberg et al., 2013), we expected subjects to be unable to report CS+/US contingencies above chance level. If high trait anxiety is associated with increased prioritization of threatening stimuli, the high-anxiety group should be able to report contingencies better than the low anxiety group. For descriptive purposes, we also assessed differences between the high and the low anxiety groups with regard to personality facets other than trait anxiety, responding to the US, and heart rate variability (HRV) using self-report questionnaires as well as electromyography (EMG) and electrocardiography (ECG).

## Materials and Methods

### Participants

The study was advertised only to females, in newspaper and on campus. We decided to include only women for the following reasons. First, anxiety disorders are much more common in women than men (e.g., Goodwin et al., 2005; Wittchen and Jacobi, 2005; Angst et al., 2015), so that females can be considered to be more at risk of suffering from anxiety than men. Second, results of a related investigation from our laboratory (Eden et al., 2014) suggested that even with a high effort in pre-screening we were unlikely to recruit a high trait-anxiety group with a balanced number of male and female participants. We thus admitted female participants only, to prevent unbalanced groups, reduce variance, and increase effect size. Potential participants received detailed information about the study procedure and general requirements (i.e., absence of magnetic material, no history of neurological or psychological disorders, maximum age of 35 years). A link was provided to an online questionnaire including the trait anxiety form (STAI-Trait) of the German Spielberger State-Trait-Anxiety-Inventory (STAI; Laux et al., 1981), and the Self-Rating Questionnaire on Hypersensitivity to Sound (GÜF; Nelting et al., 2002). STAI-Trait includes 20 statements about the general experience of anxiety, to be answered on a 4-point intensity scale. It has an excellent internal consistency (Cronbach’s α: 0.90 for men and 0.91 for women), good retest reliability (between 0.85 and 0.91 for female students after seven and more weeks), and good convergent and divergent validities (see Laux et al., 1981, for more information). Individuals with a high STAI-Trait score tend to perceive situations as threatening, and react with heightened state anxiety. GÜF, with 15 items and 4-point intensity scales, measures the degree of distress due to hearing sounds. It possesses good reliability (Cronbach’s α: 0.89; retest reliability: 0.82) and convergent validity (Nelting et al., 2002). Before completing the online survey, all 477 volunteers gave informed consent by button press. Participation in the online questionnaire was not compensated.

**Figure 1:**
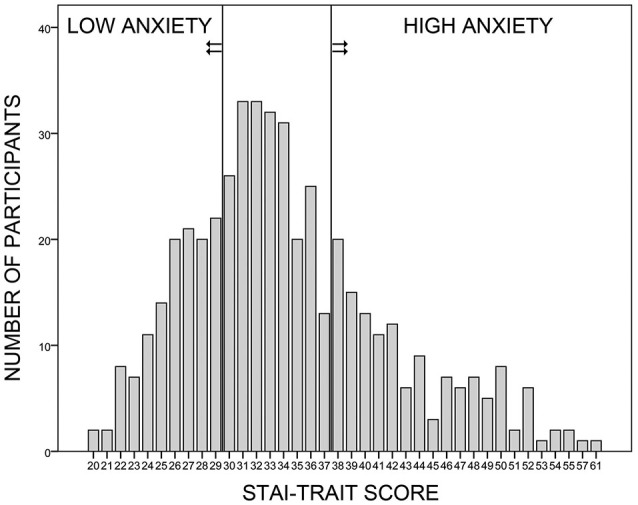
**Sample selection**. Four hundred and seventy-seven female volunteers participated in the online survey, in which they answered to the trait anxiety form of the German Spielberger State-Trait-Anxiety-Inventory (STAI; Laux et al., 1981) and to the Self-Rating Questionnaire on Hypersensitivity to Sound (GÜF; Nelting et al., 2002). Women with a trait anxiety score within the lowest or the highest quartile of the sample were recruited for the low and high anxiety groups, respectively. All participants had a GÜF score below 11 (not shown here).

Women with a STAI-Trait score within the lowest (≤29) or highest (≥38) quartile of the sample (Figure 1) and a GÜF score below 11 (thus excluding women overly sensitive to loud noise) were contacted. Those who fulfilled the general study requirements were invited. In total, 48 (25 high trait anxiety) participants took part in the study. As one participant quit during measurement, 24 participants were included in the high trait anxiety (HA) and 23 participants in the low trait anxiety (LA) group. All participants had normal or corrected-to-normal vision, and gave written informed consent to the protocol approved by the Ethics Committee of the Psychology Department at the University of Münster. Participation was compensated with 10 € per hour.

To identify differences between groups other than in trait anxiety, participants completed German versions of the state anxiety part of the STAI (STAI-State), the Anxiety Sensitivity Index (ASI; Ehlers, 1986; Reiss et al., 1986; Taylor and Cox, 1998; Fehm, 2003), and the Threatening Life-Event Scale (TLES; Brugha et al., 1985). They also reported current psychological symptomatology in the semi-structured Mini International Neuropsychiatric Interview (MINI; Ackenheil et al., 1999). The STAI-State has an excellent internal consistency (Cronbach’s α: 0.91 for men and women) and good convergent and divergent validities (see Laux et al., 1981, for more information). The ASI shows an excellent internal consistency (Cronbach’s α: between 0.93 and 0.95) and good content and convergent validities in predominantly Caucasian and African-American samples (Deacon et al., 2003; Arnau et al., 2009). In a Spanish sample, the TLES demonstrates low internal consistency (Cronbach’s α: 0.44), good to excellent retest reliability (0.86), and some evidence of convergent validity (Motrico et al., 2013). The MINI shows good to very good sensitivity and specificity of diagnosis (except for agoraphobia) and acceptable retest reliability, as revealed in French and US-American samples (Lecrubier et al., 1997). ECG was recorded during all phases of MultiCS conditioning to assess HRV.

### Stimuli

#### CSs

We used 104 photographs displaying faces of Caucasian individuals (52 male, 52 female), with neutral expression and from frontal view. Photographs came from the Karolinska Directed Emotional Faces archive (Lundqvist et al., 1998), the NimStim set of facial expressions (Tottenham et al., 2009), the FERET database of facial images (Phillips et al., 1998, 2000), and the picture pool of the Institute for Biomagnetism and Biosignalanalysis. Faces were scaled to 11 cm height and 72 pixels/inch resolution and converted to gray scale with Adobe Photoshop CS4 (Adobe, San Jose, CA, USA). Images were pseudo-randomly split into two groups, a CS+ (i.e., paired during acquisition) and a CS− group (i.e., not paired during acquisition). The assignment was flipped for every other participant. Stimulus sets did not differ in brightness or contrast (all *p*s ≥ 0.854).

#### US

A 100 ms long burst of white noise served as US. Final loudness was intended to correspond to 95 dB, a value often used in conditioning research. US loudness should be comparable across participants, but individual hearing capacities vary in the MEG surrounding. Therefore, US presentation was set to 80 dB above the individual hearing threshold, assuming an average threshold of 15 dB on both ears. Participants indeed showed a mean threshold of 14.9 dB (*SD* = 3.7, *Min* = 10, *Max* = 25 dB) on the left, and 16.4 dB (*SD* = 3.2, *Min* = 10, *Max* = 25 dB) on the right ear, confirming a resulting mean US loudness of ~95 dB. Groups did not differ in mean hearing thresholds, and consequently also not in US loudness, all *p*s ≥ 0.294. Individual sensitivity to the US was assessed by subjective ratings of unpleasantness performed after extinction. That is, participants rated US unpleasantness retrospectively on a scale ranging from *1* (not unpleasant at all) to *6* (very unpleasant). In addition, EMG (Startle-EMG) assessed reflexive fear responding to the US during acquisition.

### Experimental Procedure

**Figure 2:**
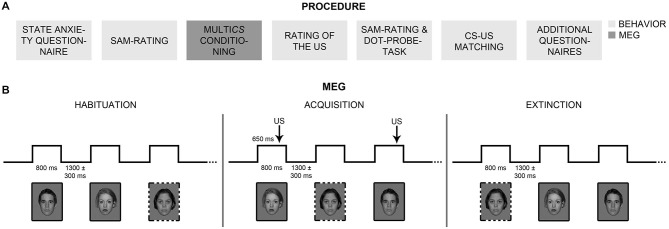
**Procedure. (A)** Participants completed behavioral measures and self-rating questionnaires before and after MultiCS conditioning outside the MEG scanner (boxes in light gray). Participants underwent MultiCS conditioning inside the MEG scanner (box in dark gray) and rated the unpleasantness of the unconditioned stimulus (US). **(B)** The MultiCS conditioning procedure consisted of three experimental phases, i.e., habituation, acquisition, and extinction. During habituation and extinction, the conditioned stimuli (CSs, 104 different neutral faces) were shown three times each in random order and without US presentation. During acquisition, half of the CSs were paired three times with the US (CS+; solid frames), while the other half remained unpaired (CS−; dotted frames).

Participants first completed the STAI-State and rated hedonic valence and emotional arousal of all CSs, using the SAM scales (Bradley and Lang, 1994; Figure 2A). Electrodes were mounted for 81-sensor electroencephalographic (EEG; not reported here), Startle-EMG, and ECG recordings. For Startle-EMG measurement, three of the six ocular EEG (easycap, Falk Minow Services) electrodes were used, positioned on the left temple and under the left eye. Electrodes for ECG measurement were placed on the left costal arch and the right curve of the neck. Impedances of all EEG, Startle-EMG, and ECG electrodes were kept below 8 kΩ, and all electrophysiological data were measured with built-in amplifiers of the MEG system. Polhemus 3Space® Fastrack measured individual head coordinates. Participants were seated in upright position inside the 275-sensor whole-head MEG system (CTF Systems Inc., BC, Canada) with radially oriented gradiometers. Initially, all CSs were shown once in randomized order, to reduce novelty effects. MEG, Startle-EMG, and ECG were measured while participants underwent MultiCS conditioning, consisting of habituation, acquisition, and extinction phases (Figure 2B). During each phase, all CSs were presented three times for 800 ms in the center of the screen (12.6° visual angle) and in pseudo-randomized order (maximally three consecutive stimuli of one CS-type). A fixation cross was presented during inter-trial intervals (ITI), jittered between 1000 and 1600 ms. During acquisition, CS+ were three times (100% contingency) paired with the US, which started 650 ms after CS+ onset, while CS− remained unpaired. Before each phase, participants were informed about appearance of faces, or of faces and sounds, but not about CS+/US contingencies. They were asked to watch the stimuli attentively and to focus gaze on the screen center. All subjects followed the instructions as monitored and ascertained via camera.

After extinction, participants retrospectively rated US unpleasantness. Subsequently, they completed a second SAM-rating, a dot-probe task, and a CS-US matching task. The order of SAM-rating and dot-probe task was balanced between participants and groups. The dot-probe task (MacLeod et al., 1986) consisted of 208 trials (set-up adapted from Stevens et al., 2009), in which a fixation cross (1500 ms duration) preceded two cues (175 ms duration), which were immediately followed by a target. Cues consisted either of two CS+ (+/+ trial), one CS+ and one CS− (+/− trial), or two CS− (−/− trial), simultaneously presented right and left of the fixation cross. Targets were arrows, pointing up or down, replacing the left or right CS. Participants pressed the 8-key (right hand) for upwards arrows, and the 2-key (left hand) for downwards arrows. Reaction times (RTs) were measured for all 52 +/+, 52 −/−, and 104 +/− trials. In 52 *congruent* +/− trials the arrow replaced the CS+ and in 52 *incongruent* +/− trials it replaced the CS−. Target location and direction were balanced across conditions. In the CS-US matching task, participants indicated for each face whether it had been paired with a loud noise during acquisition while simultaneously indicating how certain they were about their answer, using a scale ranging from −4 (*certainly without sound*) to 4 (*certainly with sound*). Finally, participants completed the ASI, TLES, and MINI questionnaires. Stimulation throughout all parts of the experiment was delivered using Presentation® (Neurobehavioral Systems). Together, the experiment took about 3.5 h.

### Analysis of General Group Comparison

#### Sample Characteristics

To confirm that the HA relative to the LA group reported heightened trait anxiety, STAI-trait values between groups were compared using a *t*-test. Other potential group differences in state anxiety (STAI-state), sensitivity to loud noise (GÜF), fear of anxiety-related symptoms (ASI), experience of threatening life-events (TLES), and psychiatric symptoms (MINI) were assessed using *t*- and *X*²-tests.

#### US Unpleasantness

A *t*-test assessed whether groups differed in subjective US unpleasantness ratings.

#### Startle-EMG

Startle responses to the US during acquisition were extracted using an in-house Matlab program. One participant (HA) could not wear an EEG net due to head size, which is why Startle-EMG data of only 46 (23 in each group) subjects were recorded and analyzed. A lab assistant renamed all response files via a coding sheet that was kept hidden until preprocessing was finished. Blind to experimental conditions, the first author scored the startle responses semi-manually using the Matlab-based software Anslab Professional (Wilhelm and Peyk, 2005), in accordance to the guidelines for human startle eyeblink electromyographic studies issued by the Society for Psychophysiological Research (Blumenthal et al., 2005). A 50 ms time-window ranging from 70 to 20 ms before US onset was chosen as baseline. Trials with artifacts were rejected and valid trials without an observable startle response were set to 0. On remaining trials, responses were scored as startle if their onset fell between 21 and 80 ms (using a startle onset criterion of twice the SD of the mean baseline activity) and if their peak was visible between 21 and 120 ms after US onset. After scoring, the first three trials of every individual were excluded to eliminate extreme responses. Magnitude and probability of startle responses were calculated for every participant. Startle magnitude describes the response strength, while startle probability equals the percentage of trials in which a response occurred, relative to all trials in which a response could have occurred. Startle-EMG magnitude was transformed from *μV* into *T*-scores (*M* = 50, *SD* = 10). Via the coding sheet, participants were reassigned to their group (HA, LA). *t*-tests were used to check whether the HA and LA groups differed in Startle-EMG magnitude or probability, which would indicate differences in unconditioned responding. We excluded one participant in the LA group from probability analysis because of extreme values. Note that throughout the analysis section, extreme values (and thus outliers) were defined as values that fell below or above twice the standard deviation of the respective group mean. Participants with extreme values (i.e., outliers) were only excluded from statistical analysis when data were normally distributed, as confirmed by visual assessment (Q-Q plots) and Kolmogorov-Smirnov tests.

#### ECG

Data from all three phases of MultiCS conditioning were extracted using an in-house Matlab program. Due to technical difficulties, data of one participant (HA) were lost, and analysis was carried out for 46 (23 HA) participants. Data files were again blinded (see Startle-EMG analysis) and preprocessed via automated batch mode of Anslab Professional (Wilhelm and Peyk, 2005), using a low pass filter of 40 Hz, a notch filter of 50 Hz, and a high pass filter of 0.5 Hz. After preprocessing, segments with an interbeat interval (IBI) above or below twice the SD of the individual mean within every phase were excluded, resulting in a rejection of 4.1% (range: 1.0–6.5%) of segments. Standard deviations of the mean interbeat interval (SDNNs) were calculated for every participant and phase, and used as a measure of heart-rate variability (HRV). Data were reassigned to the actual participant and condition via the coding sheet. An ANOVA with the factors Phase (habituation, acquisition, extinction) and Group (HA, LA) was calculated across SDNNs, to investigate whether the HA and LA group differed in HRV overall, or in any of the three phases. Three participants (2 HA) were excluded due to extreme values.

Analyses of questionnaires and second-level analyses of Startle-EMG and ECG data were conducted using SPSS Statistics (IBM). Scoring and preprocessing of Startle-EMG and ECG data was performed with the Matlab-based Anslab software (Wilhelm and Peyk, 2005).

### Analysis of Group Comparison in CS+/CS− Differentiation

#### CS-US Matching

Contingency awareness was quantified by measuring CS+ detection, using *d*′ (Green and Swets, 1966), and compared between groups via *t*-test. Awareness within each group was assessed with *t*-tests of *d*′ against the test-value *0*. Two participants (one HA) were excluded from *d*′ analysis due to extreme values. Biases in favor of “yes” (i.e., CS+) or “no” (i.e., CS−) responses were estimated using logβ (Wickens, 2002), and group differences in a potential bias were assessed via *t*-test. In analysis of response bias, five participants (three HA) with extreme values were excluded. Since log β was not normally distributed, we double-checked results by calculating nonparametric tests across all participants (i.e., not excluding outliers).

#### Ratings of Valence and Arousal

ANOVAs on valence and arousal ratings, with the factors Session (pre-, post-conditioning), CS-Type (CS+, CS−), and Group (HA, LA), assessed whether participants’ evaluative judgments changed from before to after conditioning, and whether these changes were stronger in the HA than in the LA group. Calculation of two mixed-model ANOVAs is typically adopted for investigations of group differences in subjective valence and arousal ratings (e.g., Pastor et al., 2003; Tempesta et al., 2010), and was chosen here to facilitate comparison with other studies. Subsequent item-based analyses evaluated whether potential changes in evaluative judgments (that emerged in the first analyses) were driven by the actual (i.e., CS-type) or the perceived pairing (i.e., CS-type reported in CS-US matching). Thus, the factor Report (CS+, CS−) was added to the mixed-model ANOVAs, which now included the factors Session, CS-Type, Report, and Group.

#### Dot-Probe

Trials with incorrect responses or with RTs shorter or longer than two SDs away from the individual mean, were excluded, resulting in 6.3% rejected trials. We first assessed whether the HA group showed a stronger response acceleration in congruent vs. incongruent +/− trials than the LA group, which is indicative of enhanced orienting towards CS+. An ANOVA with the factors Congruency (congruent, incongruent), Target Location (right, left), Target Direction (up, down), and Group (HA, LA) was calculated across RTs of +/− trials only. Additionally, we assessed whether the HA was faster than the LA group with increasing emotionality of the cues due to the presence of two (+/+), one (+/−), or no (−/−) CS+ stimuli. RTs of all trials were compared in an ANOVA with the factors Trial Type (+/+, +/−, −/−), Target Location (right, left), Target Direction (up, down), and Group (HA, LA). Analyses were performed once across all participants and once across right-handed participants only. Since exclusion of left-handed and ambidextrous participants did not alter the results significantly, significance values are only reported for analyses across all participants.

#### MEG Sensor Space

**Figure 3:**
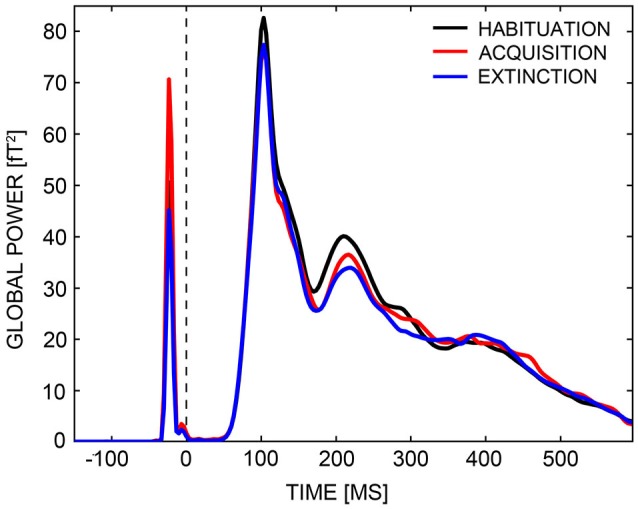
**Global power plot**. Global power of field strengths across all sensors are displayed for the habituation (black line), acquisition (red line), and extinction (blue line) phases across a time-interval, ranging from −150 ms to 600 ms. A delta peak like technical trigger artifact occurred at around −20 ms.

Visually evoked magnetic fields (VEMFs) of all participants were recorded using a 275 MEG whole-head sensor system (CTF Systems Inc., BC, Canada) with first-order axial SQUID gradiometers. Landmark coils were positioned on the two ear channels and the nasion, to register head movement during MEG recording. Head movement was quantified as maximum deviation of landmark coils from the starting position at the beginning of the session. The landmark coils of one participant (HA) became undone during measurement, and MEG data of this subject were excluded from further analysis. Movement did not exceed 0.5 cm per MEG session in all other subjects. MEG data were recorded continuously with a sampling rate of 600 Hz, across a frequency range from 0 and 150 Hz (anti-aliasing hardware filtering). The continuous data were down-sampled offline to a rate of 300 Hz, and filtered with zero-phase (forward/backward) Butterworth second-order high-pass and fourth-order low-pass filters, so that the final data only included responses between 0.1 to 48 Hz. Higher frequencies were detected to allow potential single trial analysis of induced gamma band activity which is not reported here. Data were split into single epochs encompassing an 880 ms time-window, from 200 ms before to 600 ms after CS onset. Prior testing of the MEG and video crosstalk revealed a fixed delay of 33 ms between trigger and picture onsets, so, epoch windows were adjusted for this delay. Epochs were baseline-adjusted, selecting a pre-stimulus interval of 110 ms duration (150–40 ms before CS onset) as baseline. The endpoint of the baseline interval was set to −40 ms instead of 0 ms (i.e., CS onset), because of a delta-peak like technical trigger artifact that occurred at −20 ms (Figure 3). Artifact detection and rejection was performed with an established method for statistical control of artifacts in high-density EEG/MEG data (Junghöfer et al., 2000). This procedure: (1) detects individual channel artifacts; (2) detects global artifacts; (3) replaces artifact-contaminated sensors with spline interpolation, statistically weighted on the basis of all remaining sensors; and (4) computes the variance of the signal across trials to document the stability of the averaged waveform. The rejection of artifact-contaminated trials and sensor epochs relies on the calculation of statistical parameters for the absolute measured magnetic field amplitudes over time, their standard deviation over time, as well as on the determination of boundaries for each parameter. Epochs were averaged within subjects with respect to Phase (habituation, acquisition, extinction) and CS-Type (CS+, CS−). Since CS+ faces were followed by a US, 650 ms after CS onset, CS-US associations could not have been established during acquisition within the first 650 ms of the first presentation of each CS. Thus, the first run of acquisition was discarded and differential CS+/CS− activation during acquisition was estimated using the second and third acquisition runs only. Four participants (two HA) with significantly less good trials (<2 SDs of the mean) in all experimental conditions than the remaining subjects were excluded from further analyses. Thus, analyses of MEG data were performed across 42 participants, 21 in each group. Groups did not differ in the number of good trials (total: 832 trials. HA: *M* = 784.3, *SD* = 31.5. LA: *M* = 787.8, *SD* = 34.4. *t*_(40)_ = −0.34, *p* = 0.738).

The CTF-MEG system uses radially oriented gradiometers, measuring in- and outgoing radial magnetic fields of underlying sources. The superposition of in- and outgoing fields generated by distributed neural activities often results in quite complex topographies. This complexity was reduced by transforming VEMF topographies to Root-Mean-Square (RMS) field maps of planar gradiometers with azimuthal and polar orientation. RMS field maps are always positive and—in case of a single dipolar source—maximal above the dipole location. Thus, this transformation reduces data dimensionality and allows easier comparison with MEG source-space reconstructions (e.g., L2-Minimum-Norm). Analysis of RMS values investigated whether the HA group showed a stronger acquisition and inferior extinction than the LA group, focusing on early and mid-latency MEG components (M1, M170, M2). We calculated an ANOVA with the factors Phase (habituation, acquisition, extinction), CS-Type (CS+, CS−), and Group (HA, LA) across all time-points and sensors, and looked for a significant three-way interaction in relevant time-windows. Time-windows were specified via inspection of global power plots (Figure 3), to range from 80 to 120 ms (M1), 120 to 170 ms (M170), and 170 to 260 ms (M2). A non-parametric statistical testing procedure was adopted that includes correction for multiple comparisons and is similar to the cluster mass test used for analysis of fMRI data (Maris and Oostenveld, 2007). As part of this procedure, *F*-values of sensors were summed to so-called cluster masses when the Phase × CS-Type × Group interaction exceeded a critical alpha-level of *p* = 0.05 during minimally five consecutive time-points (sensor-level criterion). Cluster masses were compared against a random permutation cluster-based alpha-level of *p* = 0.05, which was established via Monte Carlo simulations of identical analyses based on 1,000 permuted drawings of experimental conditions and participants. Only cluster masses exceeding a conservative alpha-level of *p* = 0.05 in the M1, M170, or M2 time-intervals were considered (cluster-level criterion). Thus, all reported Phase × CS-Type × Group interactions were significant to a sensor- and cluster-level of *p* < 0.05. Three-way interactions that survived the cluster test were further specified with parametric *post hoc* analyses, restricted to these clusters and time-windows of activation. For all *post hoc* tests, Bonferroni correction was used as countermeasure against inflation of Type I error (α). The Bonferroni-corrected criterion of significance is given in parenthesis.

#### MEG Source Space

Analyses of RMS values revealed the sensors that showed a significant interaction of Phase × CS-Type × Group in the M1, M170, or M2. Unless inverse modeling is applied, however, the location of the underlying current dipole(s) creating these significant effects cannot be easily inferred from the location of differentially activated sensors. We used the L2-Minimum-Norm-Estimates method (L2-MNE; Hämäläinen and Ilmoniemi, 1994) to model cortical sources of the VEMFs. When estimating distributed neural network activity, the L2-MNE inverse modeling technique is a good approach, because it does not rely on *a priori* specification of the location and/or number of current sources (Hauk, 2004). We adopted a spherical shell as source model, on which 2 (azimuthal and polar direction) × 350 dipoles were evenly allocated. We used 87% of the individually fitted head radius as source shell radius, because this value approximately matches gray matter depth. L2-MNE topographies were calculated for each individual participant and experimental condition on the basis of the averaged epochs, using a Tikhonov regularization parameter *k* of 0.2. The resulting topographies revealed neuronal source activity—independent of direction—for the different conditions. Subsequent analyses were equivalent to sensor space. Here, both sensor and source space effects are reported to provide complementary insights into conditioning-related changes in brain activity. Converging results in sensor and source space strongly foster the reliability of results and of the source estimation. Significant results that emerge in source space only do not have to support sensor space data and vice versa.

Analyses of evaluative ratings, behavior, and second-level analyses of MEG were calculated using SPSS Statistics (IBM). Preprocessing and first-level analyses of MEG data were carried out with the Matlab-based EMEGS software (Peyk et al., 2011).^1^

For all repeated-measures analyses in which the sphericity assumption was violated, Greenhouse-Geisser corrected significance values are reported. Unless otherwise noted, statistical tests were considered significant if *p* < 0.05. *Post hoc* analyses are indicated as such, and Bonferroni-corrected.

## Results

### General Group Comparison

Groups differed in trait anxiety, *t*_(36.86)_ = 24.59, *p* < 0.001, but not in age or handedness, *t*_(45)_ = −0.06 and *X*^2^_(2)_ = 3.07, *p*s ≥ 0.215 (Table 1). The HA group reported higher levels of state anxiety, *t*_(45)_ = 5.00, *p* < 0.001, greater fear of anxiety-related bodily sensations, *t*_(45)_ = 6.34, *p* < 0.001, more threatening life-events, *t*_(45)_ = 3.17, *p* = 0.003, and psychological difficulties, *X*^2^_(1)_ = 12.17, *p* < 0.001 than the LA group.

**Table 1:** **Comparison between the high (HA) and low anxiety (LA) groups**.

	High anxiety (*n* = 24) *M (SD)*	Low anxiety (*n* = 23) *M (SD)*	*t, X*^2^	*p*
STAI-Trait	**48.96 (4.12)**	**25.00 (2.36)**	**24.59**	**<0.001**
Age (in years)	23.96 (2.29)	24.00 (2.78)	−0.06	0.955
Handedness	21 right	23 right	3.07	0.215
STAI-State	**37.17 (5.51)**	**29.04 (5.63)**	**5.00**	**<0.001**
ASI	**39.75 (16.02)**	**14.04 (11.28)**	**6.34**	**<0.001**
TLES	**4.79 (1.69)**	**3.39 (1.31)**	**3.17**	**0.003**
MINI	**14 unremarkable**	**23 unremarkable**	**12.17**	**<0.001**

*To validate sample selection, HA and LA groups were compared with regard to trait anxiety scores (STAI-Trait). For characterization, groups were compared with regard to age and handedness, self-reported current anxiety (STAI-State), fear of anxiety-related bodily sensations (ASI), experience of threatening life-events (TLES), and psychological difficulties (MINI). Descriptive statistics for each measure and group, *X*^2^- and *t*-test statistics for the group comparison, and corresponding significance values are displayed. Significant comparisons are marked in bold*.

#### Unconditioned Responding

**Figure 4:**
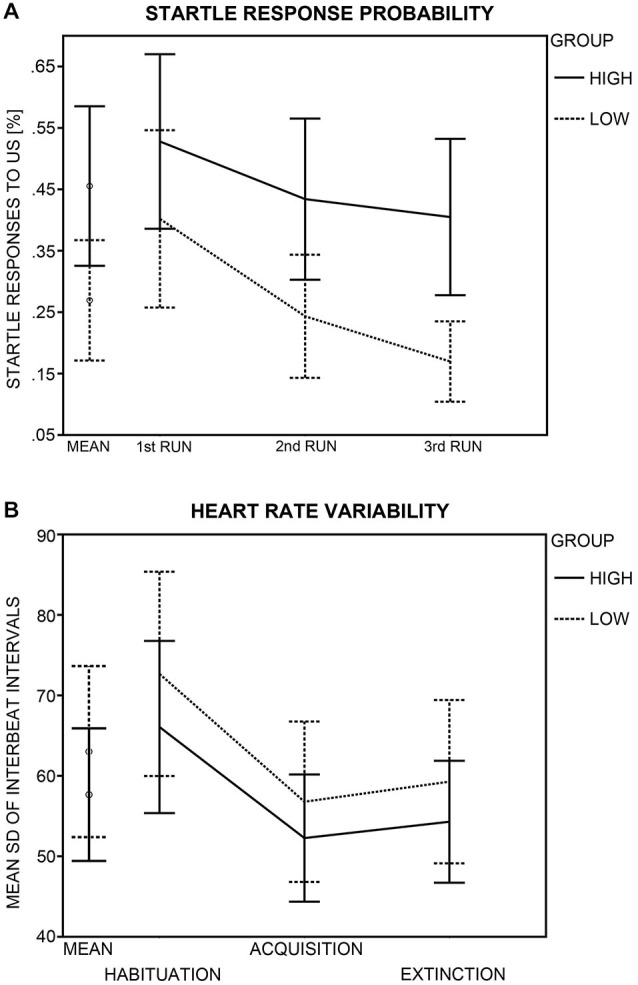
**Group differences in startle response probability and heart rate variability (HRV). (A)** Probability of a startle eye-blink response to the unconditioned stimulus (US) during acquisition (in %) is displayed for the high (crosses) and low (diamonds and solid lines) anxiety groups. Probabilities (95% confidence intervals) are shown for the mean of all acquisition runs and for each acquisition run (1st, 2nd, 3rd) separately. **(B)** HRV (95% confidence intervals) as calculated by the standard deviation of the interbeat interval (SDNN) is shown for the high and the low anxiety groups as mean across all MultiCS conditioning phases and for each phase (habituation, acquisition, extinction) separately.

HA and LA participants did not differ in subjective US-unpleasantness ratings after conditioning, *t*_(45)_ = 0.58, *p* = 0.563 (Table 2). Likewise, groups did not differ in startle response magnitude, *t*_(44)_ = 1.23, *p* = 0.226. The HA group reacted more often than the LA group to the US with a startle response, *t*_(43)_ = 2.36, *p* = 0.023 (Figure 4A). An ANOVA with the factors Run (1st, 2nd, 3rd acquisition run) and Group was calculated across startle probability, to check whether group differences in probability changed across the course of acquisition. Analysis yielded effects of Group, *F*_(1,43)_ = 5.45, *p* = 0.024, and Run, *F*_(2,86)_ = 30.88, *p* < 0.001, but not of Group × Run, *F*_(2,86)_ = 2.79, *p* = 0.084. Thus, the higher percentage of startle responses in the HA group did not change across the course of fear acquisition, but both groups habituated to the US across time. The higher startle-response frequency in the HA group was paired with an enhanced noise sensitivity, as evidenced by higher GÜF scores in the HA than in the LA group, *t*_(45)_ = 5.35, *p* < 0.001. Startle response frequency was not correlated with subjective US unpleasantness, *r*_(43)_ = 0.26, *p* = 0.080, or GÜF scores, *r*_(43)_ = 0.28, *p* = 0.063, and neither was US unpleasantness associated with GÜF, *r*_(45)_ = 0.02, *p* = 0.901 (Bonferroni-corrected *p* = 0.05/3 = 0.017).

**Table 2:** **Comparison of the high (HA) and low anxiety (LA) groups with regard to responding to the unconditioned stimulus (US)**.

	High anxiety (*n* = 24) *M (SD)*	Low anxiety (*n* = 23) *M (SD)*	*t*	*p*
US unpleasantness rating	4.30 (1.06)	4.14 (0.87)	0.58	0.563
Startle-EMG magnitude	50.92 (7.14)	48.86 (3.72)	1.23	0.226
Startle-EMG probability	**0.46 (0.30)**	0.27 (0.22)*	**2.36**	**0.023**
GÜF	**5.33 (2.43)**	**1.96 (1.85)**	**5.35**	**<0.001**

Groups were compared with regard to subjective US unpleasantness, assessed in the MEG after the extinction phase. Electromyography (EMG) measured the startle fear response to the US, and groups were compared with regard to physiological sensitivity to the US, using Startle-EMG magnitude and probability. In addition, differences in self-reported sensitivity to noise (GÜF) between groups were assessed. Descriptive statistics for each group and measure, *t*-test statistics used for group comparison, and corresponding significance values are shown. Significant comparisons are marked in bold. *For probability analysis, one participant in the LA group with extreme value was excluded.

#### Heart Rate Variability

Analysis yielded a main effect of Phase, *F*_(2,82)_ = 42.57, *p* < 0.001, but groups did not differ in HRV (Group: *F*_(1,41)_ = 0.68, *p* = 0.415; Phase × Group: *F*_(2,82)_ = 0.20, *p* = 0.782; Figure 4B). Bonferroni-corrected (*p* = 0.05/3 = 0.017) *post hoc* *t*-tests revealed HRV during habituation (*M* = 69.45, *SD* = 26.16) to be greater than during acquisition (*M* = 54.58, *SD* = 20.04) and extinction (*M* = 56.84, *SD* = 20.01), *t*s_(42)_ = 9.29 and 6.17, *p*s < 0.001, pointing to differences in vagal tone between conditioning phases.

### Group Comparison in CS+/CS− Differentiation

#### Post-Experimental Contingency Report

**Figure 5:**
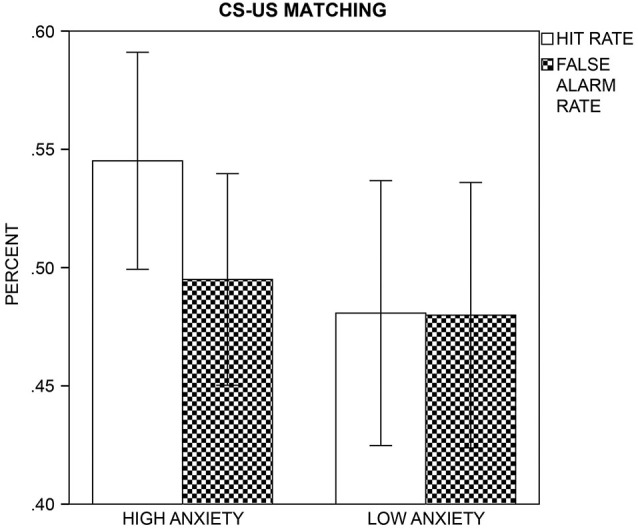
**CS-US Matching**. The mean hit (white bars) and false alarm (patterned bars) rates with a 95% confidence interval are displayed for the high and low anxiety groups.

The HA group had a better detection performance than the LA group, *t*_(43)_ = 2.41, *p* = 0.021, as d′-values indicated CS+/US contingencies above chance level in the HA, *t*_(22)_ = 3.47, *p* = 0.002, but not LA group, *t*_(21)_ = 0.09, *p* = 0.930 (Figure 5). In the HA group, contingency awareness was linearly associated with self-reported STAI-trait scores, since d′ in the HA group only correlated positively with STAI-trait, *r*_(21)_ = 0.50, *p* = 0.015 (Bonferroni-corrected *p* = 0.05/2 = 0.025). As log β values indicated, participants showed a significant bias to classify the CSs as CS+ (*M* = −0.02, *SD* = 0.04), *t*_(41)_ = −3.19, *p* = 0.003, but groups did not differ in this response bias, *t*_(40)_ = −1.12, *p* = 0.269. Nonparametric sign and Mann-Whitney U tests calculated across log β values of all participants confirmed results of the parametric analysis (one-sample sign test: *U* = −2.81, *p* = 0.005; two-sample Mann-Whitney U test: *U* = −0.77, *p* = 0.443). log β did not correlate with self-reported STAI-trait scores, all *p*s ≥ 0.111.

#### Subjective Evaluative Ratings

**Figure 6:**
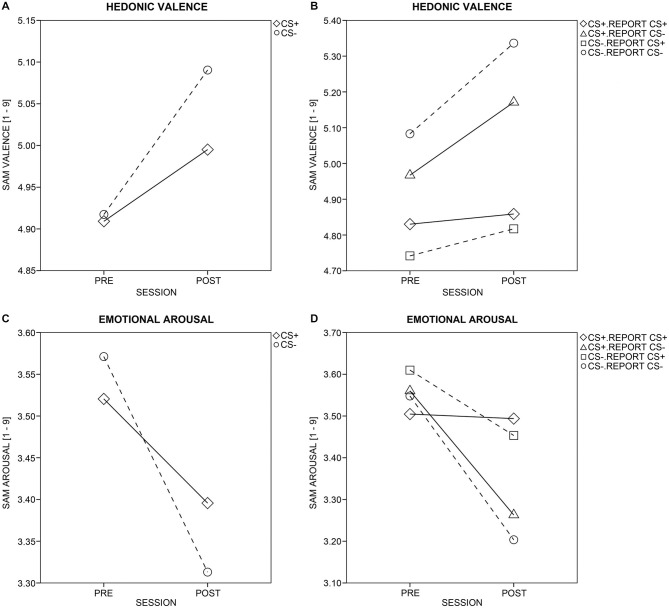
**Ratings of valence and arousal**. Changes in hedonic valence **(A)** and emotional arousal **(C)** ratings from pre- to post-conditioning are displayed for aversively paired (CS+; diamonds and solid line) and unpaired (CS−; circles and dotted line) stimuli across all participants. Changes in hedonic valence **(B)** and emotional arousal **(D)** ratings were split up for the actual and perceived/reported pairing (as reported in the CS-US matching task). Solid and dotted lines mark CS+ or CS−, respectively. Diamonds and triangles show CS+, which were reported to be CS+ or CS−, respectively, while circles and rectangles mark CS−, which were reported to be CS− or CS+, respectively.

Pleasantness ratings (Figure 6A) increased across sessions for all CSs, *F*_(1,45)_ = 5.98, *p* = 0.018, but more so for CS− than CS+ (Session × CS-Type: *F*_(1,45)_ = 4.08, *p* = 0.049). Within-subject, item-based analysis that included the reported pairing (Figure 6B) revealed that the enhanced pleasantness ratings for CS− after conditioning depended on the reported, not the actual contingency. More specifically, the four-way ANOVA yielded effects of Session, *F*_(1,45)_ = 7.88, *p* = 0.007, Report, *F*_(1,45)_ = 15.26, *p* < 0.001, CS-Type × Report, *F*_(1,45)_ = 4.88, *p* = 0.032, and Session × Report, *F*_(1,45)_ = 7.53, *p* = 0.009, but not of Session × CS-Type or Session × CS-Type × Report, *F*s_(1,45)_ = 1.28 and 0.01, *p*s ≥ 0.264. *Post hoc* *t*-tests (Bonferroni-corrected *p* = 0.05/2 = 0.025) indicated that only those CSs that were reported to be CS− in the CS-US matching, were rated as more pleasant after conditioning, compared to before, *t*_(46)_ = 4.86, *p* < 0.001. This was not the case for those CSs reported to be CS+, *t*_(46)_ = 0.76, *p* = 0.453. Importantly, the actual pairing (i.e., the CS-Type) of the CS did not influence the change in pleasantness after relative to before conditioning.

Arousal ratings (Figure 6C) decreased across sessions for all CSs, *F*_(1,45)_ = 5.46, *p* = 0.024, but more so for CS− than CS+ (Session × CS-Type: *F*_(1,45)_ = 7.23, *p* = 0.010). Within-subject, item-based analysis including the reported pairing (Figure 6D) revealed that the enhanced decrease in arousal for CS− after conditioning depended on both the actual and the reported contingency. The four-way ANOVA yielded effects of Session, *F*_(1,45)_ = 6.60, *p* = 0.014, Report, *F*_(1,45)_ = 4.25, *p* = 0.045, Session × CS-Type, *F*_(1,45)_ = 4.52, *p* = 0.039, and Session × Report, *F*_(1,45)_ = 7.48, *p* = 0.009. *Post hoc* *t*-tests (Bonferroni-corrected *p* = 0.05/4 = 0.0125) further supported that the differential decrease in perceived arousal across sessions was driven both by the actual and the reported pairing. That is, a decrease in arousal was observed for actual CS−, *t*_(46)_ = −3.43, *p* = 0.001, but not actual CS+, *t*_(46)_ = −1.75, *p* = 0.087, and for those CSs reported to be CS−, *t*_(46)_ = −3.78, *p* < 0.001, but not for those reported to be CS+, *t*_(46)_ = −0.90, *p* = 0.372.

#### Dot-Probe

Analysis across +/− trials did not reveal any orienting to CS+, as there were no effects of Congruency, all *p*s ≥ 0.310, but only of Target Direction, and Target Location × Target Direction, *F*s_(1,45)_ = 4.51 and 69.46, *p*s ≤0.039. Participants responded faster with their right (*M* = 483.63, *SD* = 48.38) than left (*M* = 490.08, *SD* = 47.78) hand, and this was true for right, but reversed for left visual-field presentations of the target.

Next, we tested whether effects of Congruency emerged on contingency-aware trials only, and performed analogous analyses across those trials for which contingencies of both faces were correctly reported. Amongst effects of Target Direction, *F*_(1,33)_ = 6.51, *p* = 0.016, Target Location × Target Direction, *F*_(1,33)_ = 20.30, *p* < 0.001, Target Location × Target Direction × Group, *F*_(1,33)_ = 8.12, *p* = 0.007, an interaction of Congruency × Target Location emerged, *F*_(1,33)_ = 7.29, *p* = 0.011. The interaction resulted from faster reactions to incongruent (*M* = 476.79, *SD* = 38.82) than to congruent (*M* = 488.63, *SD* = 51.11) trials with left visual-field presentations of the target, but faster reactions to congruent (*M* = 484.11, *SD* = 51.88) than to incongruent (*M* = 497.40, *SD* = 51.90) trials with right visual-field presentations of the target. In both cases, participants reacted fastest when the right cue face was a CS+.

Analysis including Trial Type did not reveal any influence of cue emotionality, when calculated across all trials, or contingency-aware trials only, all *p*s ≥ 0.254.

#### M1 Sensor Space

**Figure 7:**
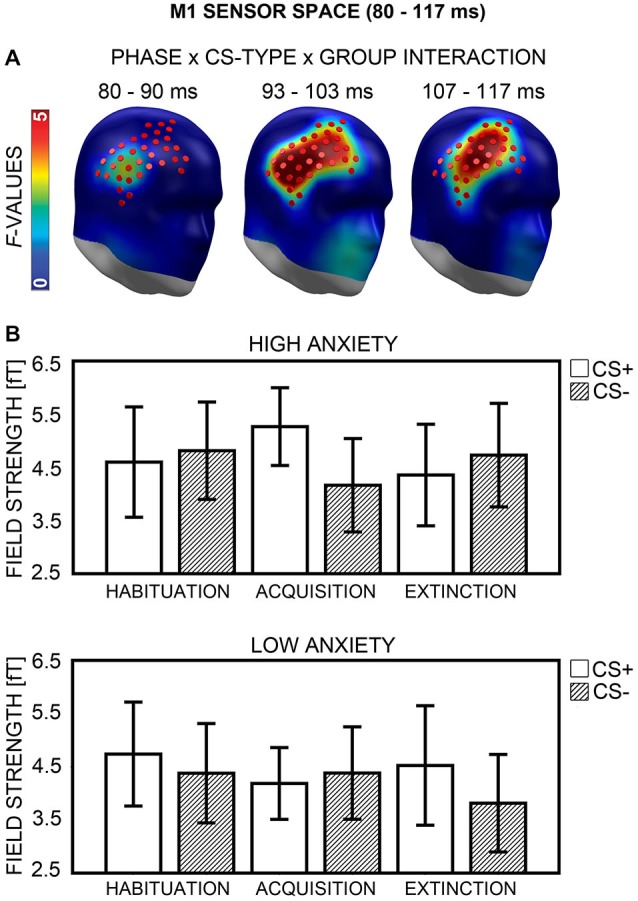
**Visualization of the M1 sensor space effect (80–117 ms). (A)** *F*-values for the Phase × CS-Type × Group interaction calculated for the M1 time-interval are projected onto right frontal view standard heads. Red disks visualize the sensor locations used for *post hoc* statistical analyses. **(B)** Mean field strengths (95% confidence intervals) are shown for paired (CS+; white bars) and unpaired (CS−; striped bars) stimuli for the high and low anxiety groups and the three conditioning phases.

A Phase × CS-Type × Group interaction was observed in a right fronto-temporal sensor cluster (Figure 7A) between 80 and 117 ms, with a peak at 103 ms after CS onset. This interaction indicated that the HA and LA group differed in CS+/CS− processing across phases (Figure 7B). To break up the three-way interaction, ANOVAs with the factors CS-Type and Group were calculated separately for each phase (Bonferroni-corrected significance: *p* = 0.05/3 = 0.017). There was no significant interaction of CS-Type × Group during habituation, *F*_(1,40)_ = 3.03, *p* = 0.089. During acquisition, the ANOVA yielded a significant two-way interaction, *F*_(1,40)_ = 8.57, *p* = 0.006. *Post hoc* *t*-tests (Bonferroni-corrected *p* = 0.05/2 = 0.025) showed enhanced field strength within the HA group for CS+ (*M* = 5.27, *SD* = 1.61) relative to CS− (*M* = 4.16, *SD* = 1.93), *t*_(20)_ = 3.72, *p* = 0.001, while the LA group revealed no such difference in CS+ (*M* = 4.19, *SD* = 1.48) vs. CS− (*M* = 4.38, *SD* = 1.91) field strength, *t*_(20)_ = −0.59, *p* = 0.560. A significant CS-Type × Group interaction again emerged during extinction, *F*_(1,40)_ = 6.48, *p* = 0.015. Here, the LA group showed enhanced field strength for CS+ (*M* = 4.52, *SD* = 2.47) relative to CS− (*M* = 3.81, *SD* = 2.01), *t*_(20)_ = 2.19, *p* = 0.041, but the difference did not exceed Bonferroni-corrected significance (*p* = 0.05/2 = 0.025). The HA group showed no significant difference in CS+ (*M* = 4.36, *SD* = 2.10) vs. CS− (*M* = 4.73, *SD* = 2.14) field strength *t*_(20)_ = −1.36, *p* = 0.190 during extinction.

The second part of analyses investigated whether the group differences in CS+ field strength observed during acquisition and extinction were in any way related to enhanced contingency awareness in the HA group. First, two correlational analyses were performed (Bonferroni-corrected *p* = 0.05/2 = 0.025). Differences between CS+ and CS− field strength during acquisition and extinction were computed for all participants, and correlated with the sensitivity measure d′. No significant association emerged for extinction, *r*_(38)_ = −0.11, *p* = 0.486, but there was a positive correlation between d′ and the difference between CS+ and CS− field strength during acquisition, *r*_(38)_ = 0.33, *p* = 0.039. However, the correlation did not exceed Bonferroni-corrected significance (*p* = 0.025). Next, two item-based ANOVAs were carried out (Bonferroni-corrected *p* = 0.05/2 = 0.025), for acquisition and extinction phases, now containing the factors CS-Type, Group, and Report (i.e., CS-type reported in the CS-US matching task). Analyses yielded no main effect and no interactions for Report, all *p*s ≥ 0.288.

#### M1 Source Space

**Figure 8:**
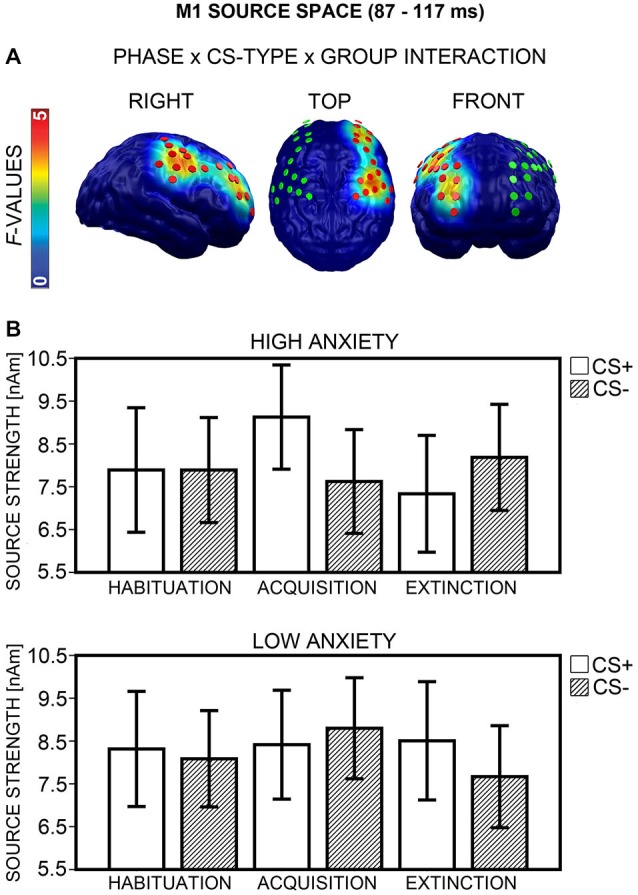
**Visualization of the M1 source space effect (87–117 ms). (A)** *F*-values for the Phase × CS-Type × Group interaction calculated for the M1 time-interval are projected onto standard brains, displayed from right, top, and frontal view. Red disks visualize the dipole locations used for *post hoc* statistical analyses, while green disks visualize the dipole locations of the mirror-symmetric left-hemispheric cluster. **(B)** Mean source strengths (95% confidence intervals) are shown for paired (CS+; white bars) and unpaired (CS−; striped bars) stimuli for the high and low anxiety groups and the three conditioning phases.

Analysis yielded a Phase × CS-Type × Group interaction in an area encompassing the right dorsolateral prefrontal (dlPFC) and premotor cortices (Figure 8A, left), which emerged during an 87 and 117 ms interval (peak: 103 ms). Separate ANOVAs for each phase showed a significant interaction of CS-Type × Group after Bonferroni correction (*p* < 0.017) during acquisition, *F*_(1,40)_ = 11.58, *p* = 0.002, and extinction, *F*_(1,40)_ = 8.08, *p* = 0.007, but not habituation, *F*_(1,40)_ = 0.24, *p* = 0.629. The direction of interactions (Figure 8B) corresponded to M1 sensor space (cf. Figure 7B). *Post hoc* *t*-tests (Bonferroni-corrected *p* = 0.05/2 = 0.025) revealed a significant difference between CS+ and CS− activation in the HA, *t*_(20)_ = 3.74, *p* = 0.001, but not in the LA group, *t*_(20)_ = −1.00, *p* = 0.327, during acquisition. CS+/CS− differences during extinction did not survive Bonferroni-correction in either group, all *p*s ≥ 0.051. Again, a correlation between higher CS+ than CS− activation during acquisition and individual d′ score was evident, *r*_(37)_ = 0.36, *p* = 0.025, now marginally significant after Bonferroni-correction, when an outlier with extreme values was excluded. The correlation indicated that the greater the neuronal enhancement of CS+ relative to CS− during acquisition, the more the individual was capable of differentiating between CS+ and CS− after conditioning in the CS-US matching task. There was no correlation during extinction, *p* ≥ 0.372. Item-based analyses again did not show any main effect or interaction of the factor Report during acquisition or extinction, all *p*s ≥ 0.104. Thus, for the M1 time-interval, effects in source space supported effects in sensor space.

##### Hemispheric asymmetry

The regional extent of the Phase × CS-Type × Group interaction (Figure 8A) suggested an involvement of the right, but not left hemisphere in differential CS+/CS− processing. An ANOVA with the factors Phase × CS-Type × Group × Hemisphere (right, left) on the data from the right and a mirror-symmetric left-hemispheric cluster (Figure 8A, middle and right), supported this impression, as it yielded a significant four-way interaction, *F*_(2,80)_ = 6.74, *p* = 0.003. Analysis of the left cluster alone failed to reveal a Phase × CS-Type × Group interaction, *F*_(2,80)_ = 1.24, *p* = 0.296.

#### M170 Sensor Space

**Figure 9:**
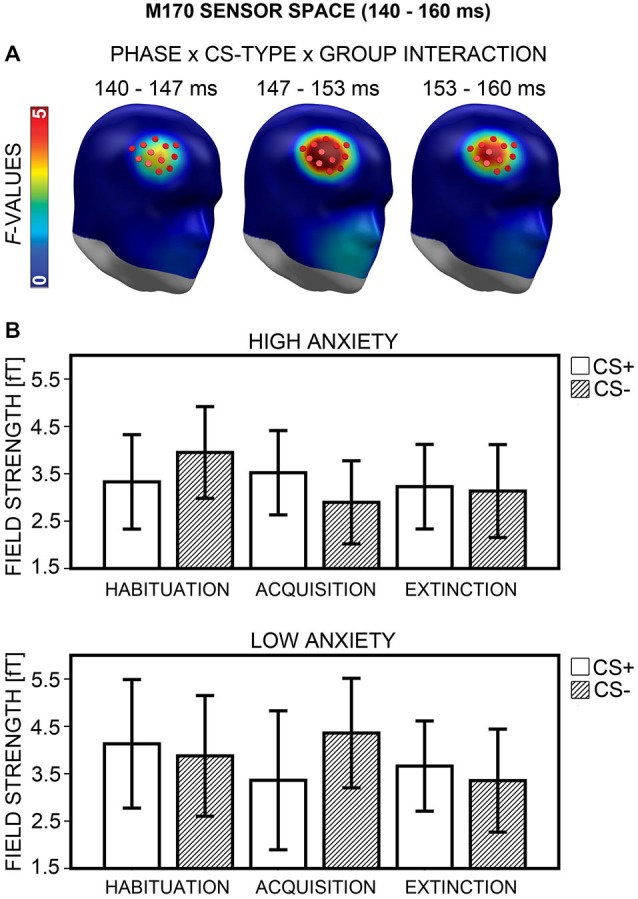
**Visualization of the M170 sensor space effect (140–160 ms). (A)** *F*-values for the Phase × CS-Type × Group interaction calculated for the M170 time-interval are projected onto right frontal view standard heads. Red disks visualize the sensor locations used for *post hoc* statistical analyses. **(B)** Mean field strengths (95% confidence intervals) are shown for paired (CS+; white bars) and unpaired (CS−; striped bars) stimuli for the high and low anxiety groups and the three conditioning phases.

A Phase × CS-Type × Group interaction emerged in a right fronto-temporal cluster, between 140 and 160 ms (Figure 9A), and peaking at 150 ms after CS onset. ANOVAs with the factors CS-Type and Group, calculated separately for each phase, yielded a significant CS-Type × Group interaction after Bonferroni correction (*p* < 0.017) for acquisition, *F*_(1,40)_ = 8.73, *p* = 0.005, but not for habituation, *F*_(1,40)_ = 3.49, *p* = 0.069, and extinction, *F*_(1,40)_ = 0.19, *p* = 0.664. During acquisition the same pattern was observed as for the acquisition phase in the M1 time-interval: the HA group showed stronger field strength for CS+ (*M* = 3.52, *SD* = 1.96) than for CS− (*M* = 2.90, *SD* = 1.93), while the LA group revealed the opposite: weaker field strength for CS+ (*M* = 3.36, *SD* = 3.22) than for CS− (*M* = 4.36, *SD* = 2.54; Figure 9B). However, neither the difference in the HA, *t*_(20)_ = 1.83, *p* = 0.082, nor in the LA group, *t*_(20)_ = −2.32, *p* = 0.031, reached significance in Bonferroni-corrected *post hoc* *t*-tests. Correlational analyses revealed a positive association between the CS+ vs. CS− field strength difference during acquisition and the sensitivity measure d′, but this association was not significant, even when excluding an outlier with extreme value, *r*_(37)_ = 0.25, *p* = 0.130. Item-based analysis did not yield any effect of contingency awareness during acquisition, all *p*s ≥ 0.184.

#### M170 Source Space

**Figure 10:**
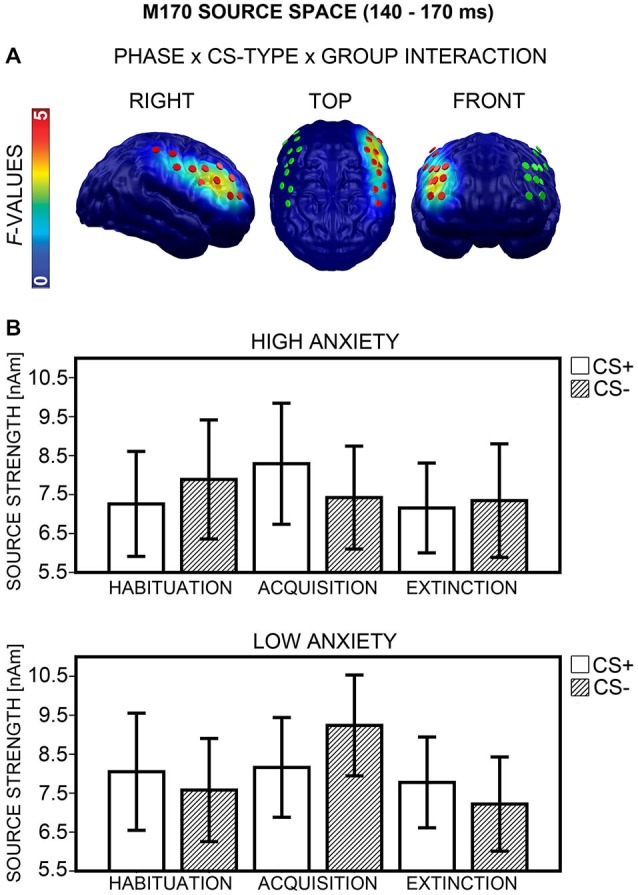
**Visualization of the M170 source space effect (140–170 ms). (A)** *F*-values for the Phase × CS-Type × Group interaction calculated for the M170 time-interval are projected onto standard brains, displayed from right, top, and frontal view. Red disks visualize the dipole locations used for *post hoc* statistical analyses, while green disks visualize the dipole locations of the mirror-symmetric left-hemispheric cluster. **(B)** Mean source strengths (95% confidence intervals) are shown for paired (CS+; white bars) and unpaired (CS−; striped bars) stimuli for the high and low anxiety groups and the three conditioning phases.

A Phase × CS-Type × Group interaction emerged in the right dlPFC (Figure 10A, left) between 140 to 170 ms (peak: 153 ms). ANOVAs with the factors CS-Type and Group, calculated separately for each phase, showed significant CS-Type × Group interactions after Bonferroni correction (*p* < 0.017) again during acquisition, *F*_(1,40)_ = 11.04, *p* = 0.002, but not during habituation, *F*_(1,40)_ = 4.74, *p* = 0.035, and extinction, *F*_(1,40)_ = 1.67, *p* = 0.204. The direction of interaction (Figure 10B) was equivalent to sensor space (cf. Figure 9B). *Post hoc* *t*-tests (Bonferroni-corrected) showed that the differential CS+/CS− activation during acquisition was marginally significant in the HA group, *t*_(20)_ = 2.41, *p* = 0.026, and in the LA group, *t*_(20)_ = −2.33, *p* = 0.030. There was no significant correlation between d′ and differential CS+/CS− activation during acquisition, *p* ≥ 0.395, and item-based analysis showed no effects of Report, all *p*s ≥ 0.134. Thus, contingency awareness did not influence differential M170 activity during acquisition. For the M170 time-interval, effects in source space supported effects in sensor space.

##### Hemispheric asymmetry

Analysis across the right and a mirror-symmetric left-hemispheric cluster (Figure 10A, middle and right) showed significant effects only in the right, not in the left hemisphere (Phase × CS-Type × Group × Hemisphere: *F*_(2,80)_ = 7.24, *p* = 0.002).

#### M2 Sensor and Source Space

There were no significant effects during the M2 time-interval (i.e., 170–260 ms) in sensor or in source space.

## Discussion

We investigated effects of trait anxiety on emotional learning in an ambiguous and cognitively challenging classical conditioning setting using neurophysiological, evaluative, and behavioral measures. Women scoring high or low in trait anxiety underwent MultiCS conditioning, with neutral faces as CSs, and a loud noise as US. Post-experimental contingency reports revealed that the HA group was better than the LA group at detecting CS+, and detection performance increased linearly with self-reported trait anxiety scores. Whole-head MEG showed that the HA group revealed more neuronal activation to CS+ than to CS− stimuli, which was not the case for the LA group. This pattern was observed in the M1 and M170 time-intervals, during the acquisition of CS+/US contingencies. Source modeling indicated an involvement of the right dlPFC and premotor cortex. For the M1, differential CS+/CS− activation during learning was associated with CS+ detection performance measured after learning. However, trait anxiety did not affect behavioral measures of selective attention to aversively paired faces (dot-probe), nor evaluative judgments (SAM-ratings). Although groups did not differ in their evaluation of US unpleasantness or startle-response magnitude, the HA group revealed higher self-reported noise sensitivity and more fear startle responses to the US than the LA group. Groups did not differ in heart-rate variability across phases.

### No Effect of Trait Anxiety on Evaluative Ratings and Behavioral Orienting

After MultiCS conditioning, unpaired faces were rated as more pleasant and less arousing than aversively paired faces, relative to the before-conditioning baseline. Such changes in evaluative ratings that depend on CS-type are consistent with data from fear-conditioning studies with only two CSs, and could be interpreted as signaling successful fear conditioning. However, grouping items by report (i.e., by the CS-type reported in the CS-US matching task) revealed that changes in evaluative judgments followed the reported rather than the actual contingency—at least where pleasantness is concerned. This dependence of evaluative judgments on deliberate categorization was observed in the HA and in the LA group. This implicates, on the one hand, that evaluative ratings without information about reported contingencies are not optimal to assess the success of fear conditioning manipulations. Other more implicit measures, such as RT, could provide a less cognitively biased estimation of fear learning (Pleyers et al., 2007). On the other hand, the result suggests that if effects of trait anxiety on evaluative judgments are observed, they are closely connected not only to individual differences in judging CS value, but also in estimating the probability of US occurrence (i.e., US expectancy).

The dot-probe task has shown selective attention to threat stimuli in clinically or subclinically anxious groups (e.g., Salemink et al., 2007; Stevens et al., 2009, 2011). There are different explanations for the fact that we found no behavioral attentional bias to CS+ in any group. First, our negatively conditioned neutral faces might have less emotionality and, thus, less “cueing power” than affective stimuli such as emotional faces or scenes, often used in dot-probe experiments. Cueing power might be further reduced when the task is administered after an extinction phase. Second, conditioning with multiple CSs and few learning trials may establish relatively volatile CS+/US associations. A strong Simon effect (Simon and Rudell, 1967; Simon, 1990), due to the (in)congruence of target and response-button position, can easily overshadow a small congruency effect. A third factor concerns the type of dot-probe task. In the standard version, participants simply decide on the occurrence of a probe. In our version, participants had to discriminate between two probes (i.e., upwards and downwards arrows), which might have further obstructed the detection of an attentional bias. It has been suggested before that detection might be superior to discrimination in revealing effects of trait anxiety on behavioral orienting towards threat-relevant stimuli (Salemink et al., 2007).

### Trait Anxiety, Contingency Awareness, and dlPFC Recruitment

In previous MultiCS conditioning studies (e.g., Steinberg et al., 2013), with a similar number of CSs and acquisition trials, participants could not report CS+/US contingencies. In the present study, the LA group did not perform above chance level, but the HA group was clearly better at detecting CS+. This could be attributed to an increased acquisition and/or a decreased extinction (i.e., a better memory) of CS+/US associations. Following Caulfield et al. (2013), it seems more likely that the good performance of the HA group was driven by differences in acquisition and not in extinction of CS+/US associations. Caulfield et al. (2013) reported enhanced acquisition of eye-blink conditioning in trait-anxious subjects and suggested that the highly anxious participants perceive their environment differently, and focus more on detecting (potentially threat-signaling) contingencies. However, other fear conditioning studies reported that trait-anxious subjects acquire CS+/US contingencies less well than non-anxious participants (e.g., Chan and Lovibond, 1996; Baas et al., 2008).

Certain characteristics of our MultiCS conditioning task might have potentiated differences between the two groups in terms of fear acquisition and contingency awareness. The multitude of visually similar CSs and the few repetitions obviously made it hard to correctly report CS+/US contingencies. One option is that the better participants were at differentiating the various faces, the better they performed at the CS-US matching task. Interestingly, enhanced contingency awareness has been associated with increased working-memory capacity, and greater attentional control (Cosand et al., 2008; Baas, 2013). Highly (socially) trait-anxious participants showed enhanced visual working-memory capacity, unless confronted with distractors (Moriya and Sugiura, 2012). Thus, one could speculate that the HA group either possessed superior visual working-memory capacity, or attributed more of this capacity to the differentiation of potentially threat- or safety-signaling faces, which facilitated fast fear acquisition and increased contingency awareness. This interpretation is supported by the positive association between individual CS+ detection performance and enhanced CS+ relative to CS− activation, observed during fear acquisition in the right dlPFC and premotor cortex in the M1 time interval. Indeed, activity in the middle frontal gyrus has been linked to explicit contingency awareness on a trial-by-trial basis (Carter et al., 2006) and activity in the right middle and inferior PFC has been related to visual working memory for objects, such as faces (see Ungerleider et al., 1998, for a review). Furthermore, Pessoa (2010) suggested that involvement of the dlPFC—especially in tasks targeting working memory or response inhibition—is indicative of an integration of emotion and cognition. Such an integration was observed by Gray et al. (2002), who reported that both dlPFC activation and behavioral performance during a working-memory task (i.e., three-back) were modulated by the induction of positive, neutral, and negative emotional states. As in the present study, they found a linear (though negative) relationship between the degree to which dlPFC activity and behavioral accuracy were affected by the induced emotion state. In sum, although acquisition of explicit CS+/US associations is difficult in MultiCS conditioning, high trait anxiety enhances CS+ detection on a behavioral and neuronal level, probably by increasing the amount of (visual) working memory available for learning. Differential dlPFC activation during the M1 was related to contingency awareness on a between-subjects (i.e., correlational), but not a within-subject level (i.e., item-based analysis by report). More activation for CS+ than for CS− during the M1 may thus be a necessary, but not a sufficient condition for subsequent CS+ behavioral detection. This fits with the interpretation that differential dlPFC activation during the M1 and behavioral contingency report are not directly related, but that their association is mediated by a common underlying variable, such as trait anxiety or individual differences in visual working-memory. Interestingly, HRV was lower during fear acquisition and extinction than during habituation. A reduced HRV has been associated with increases in cognitive functioning (e.g., Heslegrave et al., 1979), but also with enhanced state, trait, or clinical anxiety (see Friedman, 2007, for a review), which further supports our interpretation of both cognition and anxiety-related effort involved in MultiCS conditioning.

The early latency (80–117 ms), the lateralization to the right hemisphere, and the localization of the effect of trait anxiety on neuronal CS+/CS− differentiation all agree with a dysregulation of bottom-up and top-down attention processes in trait anxiety (Bishop, 2007; Eysenck et al., 2007). As anxiety enhances attention to potentially threatening stimuli (Eysenck et al., 2007), trait-anxious participants might make increasing use of a bottom-up system that enables detection of behaviorally relevant stimuli, located in right temporoparietal and inferior frontal cortex (Corbetta and Shulman, 2002). Enhanced bottom-up processing might interfere with, or even obstruct, more detailed top-down processing at later stages. For example, high-anxious individuals revealed an enhanced P1, but a decreased early posterior negativity and fronto-central positivity when viewing fearful vs. neutral faces (Holmes et al., 2008). Here, the HA group showed enhanced activation to CS+ than to CS− during the M1 and M170, and thus before 170 ms, which was not present in the LA group. No differences in neuronal activation across phases and CS-types emerged between groups during the M2 (>170 ms).

### Enhanced Conditionability, Reduced Fear Inhibition, and Other Vulnerability Factors

The findings that the HA showed better contingency awareness than the LA group, and enhanced neuronal activation towards CS+ in the M1 and M170 time-intervals during fear acquisition, support the idea that enhanced conditionability (e.g., Orr et al., 2000) is a vulnerability factor for the development of anxiety disorders. This is in line with other classical conditioning studies in which fear memory was more easily acquired (e.g., Orr et al., 2000; Holloway et al., 2012; Caulfield et al., 2013; Mosig et al., 2014) and more resistant to extinction (e.g., Pitman and Orr, 1986; Orr et al., 2000; Peri et al., 2000) in clinically and subclinically anxious than in non-anxious individuals. Our present study extends such findings, showing that high trait anxiety facilitates fear acquisition of multiple CS+/US associations, even in cognitively challenging and ambiguous situations, and with strongly reduced contingency awareness.

At first glance, our main results seem to argue against the idea that reduced fear inhibition (i.e., a lack of inhibitory responding towards the safety stimulus) mediates the development of anxiety disorders in anxiety-vulnerable individuals (e.g., Davis et al., 2000). Reduced fear inhibition has been shown with clinically and subclinically anxious subjects (e.g., Grillon and Morgan, 1999; Gazendam et al., 2013) and supported by a meta-analysis on classical conditioning studies with anxiety patients (cf., Lissek et al., 2005). Although our present results rather support enhanced conditionability than reduced fear inhibition in anxiety vulnerability, we propose that both mechanisms may play a role in decreasing an individual’s resistance to clinical anxiety. The two theories might be reconciled in three different ways. First, enhanced conditionability and reduced fear inhibition could represent vulnerability factors for different types of anxiety disorders. As suggested by Mosig et al. (2014), reduced fear inhibition to the safety stimulus during fear extinction mainly characterizes post-traumatic stress disorder (PTSD), whereas specific phobias, such as spider phobia, seem associated with enhanced fear acquisition. Second, both mechanisms could have a differential impact during the course of fear acquisition. For example, anxiety-vulnerable relative to invulnerable individuals could acquire CS+/US contingencies more readily during initial learning instances, and such enhanced responding towards aversively paired stimuli could facilitate fear transfer to unpaired stimuli. Although reasonable, this hypothesis remains to be tested by future experimental studies on the time course of fear acquisition. Third, results concerning the effect of trait anxiety on fear conditioning might turn out to be influenced by other variables, which cannot yet all be assessed in one study. For example, certain variations in the serotonin transporter gene 5-HTT have been associated with enhanced conditionability, and a greater likelihood of developing PTSD after trauma (see Lonsdorf and Kalisch, 2011, for a comprehensive review). Thus, the fact that some studies found a link between trait anxiety and enhanced fear acquisition while other studies failed to show such association might result from different distributions of 5-HTTLPR s-allele carriers in the sample. We thus cannot rule out that enhanced fear acquisition in the HA group compared to the LA group in our study was not solely elicited by differences in trait anxiety, but also by differences in any other personality measure (e.g., state anxiety), or by genetic differences. Note that the differences between our groups might be particularly strong, because we tested female participants only. Previous studies using skin conductance responses showed that fear acquisition was stronger in women than in men, both for healthy individuals (Guimarães et al., 1991) as for PTSD patients (Inslicht et al., 2013). However, this effect is not consistently observed; some researchers found no differences between women and men in skin-conductance measures during fear acquisition (Zorawski et al., 2005), others even observed stronger fear acquisition in men than in women (Milad et al., 2006). The picture probably is even more complex, given the findings of complex interactions between sex, trait vulnerability, and context (Grillon, 2002), or sex and stress (Jackson et al., 2006). Future research should explore the differential influence and the interaction of such individual-difference variables.

### Trait Anxiety and Unconditioned Responding

The HA and LA groups had similar subjective US unpleasantness ratings, although the HA group revealed larger general noise sensitivity (i.e., GÜF scores) than the LA group. These results are not contradictory, when considering the populations and the range of the GÜF. The GÜF was originally designed to measure enhanced noise sensitivity in clinical populations, such as tinnitus patients, and ranges from *0* as a minimum to *45* as a maximum score (Nelting et al., 2002). Scores below *11* are still considered to indicate mild sensitivity to loud noise. Thus, although the HA was more noise-sensitive than the LA group, the mean scores of both groups fell into the same category, and were well below the cut-off point that marks the transition to more severe noise sensitivity.

Consistent with similar group ratings of US unpleasantness, trait anxiety did not affect the strength of the startle-EMG response to the US. The HA group, however, showed more startle responses to the US than the LA group. Consistent with a dual system of startle response probability and amplitude (cf., Blumenthal and Berg, 1986; Blumenthal, 1996), trait anxiety seems to enhance threat detection, but does not affect threat identification. An interesting connection to consider in future studies is the proposed relationship between tonic modulations of startle reactivity and the bed nucleus of the stria terminalis (BNST; e.g., Davis et al., 1997; Davis, 1998; see Vaidyanathan et al., 2009, for an overview). Indeed, highly anxious individuals elicit increased BNST activity, especially during proximity to threat, which was interpreted as exaggerated environmental-threat monitoring in trait anxiety (Somerville et al., 2010).

### Limitations

To reduce variance and to increase effect sizes in this first MultiCS conditioning study, in which we explored effects of individual anxiety on affective learning, we included female participants only. Thus, results can hardly be generalized to a population of men, as several studies have shown considerable differences between the sexes in fear conditioning (e.g., Milad et al., 2006; Inslicht et al., 2013). Obviously, we cannot say anything about differential influences of participant gender, as we recruited females only.

## Conclusions and Future Directions

In conclusion, our results show that females with high anxiety vulnerability show anomalies in MultiCS conditioning, even without a clinical manifestation of anxiety. These anomalies point to enhanced fear acquisition in highly trait-anxious females, and relate high trait anxiety with increases in visual working-memory capacity, threat monitoring and detection, and with bottom-up compared to top-down attention processes. Our findings are in line with theories of enhanced conditionability (e.g., Otto et al., 2007; Caulfield et al., 2013) in trait anxiety. MultiCS conditioning seems a good tool for investigating individual differences in fear acquisition, because it relies on ambiguous CS+/US associations and challenges the resolving power of human fear learning. In addition, it allows for a precise investigation of the course of acquisition and/or extinction, and for a differentiation of effects of emotion (*de facto*) and cognition (reported pairing). This holds promise for future basic and clinical anxiety research.

## Conflict of Interest Statement

The authors declare that the research was conducted in the absence of any commercial or financial relationships that could be construed as a potential conflict of interest.

## Footnotes

^1^www.emegs.org

## References

[B1] Ackenheil M. Stotz G. Dietz-Bauer R. Vossen A. (1999). M.I.N.I.–Mini International Neuropsychiatric Interview. German Version 5.0.0, DSM-IV. München: Psychiatrische Universitätsklinik München.

[B2] Angst J. Paksarian D. Cui L. Merikangas K. R. Hengartner M. P. Ajdacic-Gross V.. (2015). The epidemiology of common mental disorders from age 20 to 50: results from the prospective Zurich cohort study. Epidemiol. Psychiatr. Sci. 1–9. 10.1017/s204579601500027x 25802979 PMC6998675

[B3] Arnau R. C. Broman-Fulks J. J. Green B. A. Berman M. E. (2009). The anxiety sensitivity index—revised confirmatory factor analyses, structural invariance in caucasian and African American samples and score reliability and validity. Assessment 16, 165–180. 10.1177/1073191108328809 19104031

[B4] Arnaudova I. Krypotos A.-M. Effting M. Boddez Y. Kindt M. Beckers T. (2013). Individual differences in discriminatory fear learning under conditions of ambiguity: a vulnerability factor for anxiety disorders? Front. Psychol. 4:298. 10.3389/fpsyg.2013.00298 23755030 PMC3664781

[B5] Baas J. M. P. (2013). Individual differences in predicting aversive events and modulating contextual anxiety in a context and cue conditioning paradigm. Biol. Psychol. 92, 17–25. 10.1016/j.biopsycho.2012.02.001 22342768

[B6] Baas J. M. P. van Ooijen L. Goudriaan A. Kenemans J. L. (2008). Failure to condition to a cue is associated with sustained contextual fear. Acta Psychol. (Amst). 127, 581–592. 10.1016/j.actpsy.2007.09.009 18048004

[B7] Beckers T. Krypotos A.-M. Boddez Y. Effting M. Kindt M. (2013). What’s wrong with fear conditioning? Biol. Psychol. 92, 90–96. 10.1016/j.biopsycho.2011.12.015 22223096

[B8] Berggren N. Derakshan N. (2013). Attentional control deficits in trait anxiety: why you see them and why you don’t. Biol. Psychol. 92, 440–446. 10.1016/j.biopsycho.2012.03.007 22465045

[B9] Bishop S. J. (2007). Neurocognitive mechanisms of anxiety: an integrative account. Trends Cogn. Sci. 11, 307–316. 10.1016/j.tics.2007.05.008 17553730

[B10] Blumenthal T. D. (1996). Inhibition of the human startle response is affected by both prepulse intensity and eliciting stimulus intensity. Biol. Psychol. 44, 85–104. 10.1016/0301-0511(96)05214-3 8913523

[B11] Blumenthal T. D. Berg W. K. (1986). Stimulus rise time, intensity and bandwidth effects on acoustic startle amplitude and probability. Psychophysiology 23, 635–641. 10.1111/j.1469-8986.1986.tb00682.x 3823338

[B12] Blumenthal T. D. Cuthbert B. N. Filion D. L. Hackley S. Lipp O. V. van Boxtel A. (2005). Committee report: guidelines for human startle eyeblink electromyographic studies. Psychophysiology 42, 1–15. 10.1111/j.1469-8986.2005.00271.x 15720576

[B13] Bradley M. M. Lang P. J. (1994). Measuring emotion: the self-assessment Manikin and the semantic differential. J. Behav. Ther. Exp. Psychiatry 25, 49–59. 10.1016/0005-7916(94)90063-9 7962581

[B14] Bröckelmann A.-K. Steinberg C. Dobel C. Elling L. Zwanzger P. Pantev C.. (2013). Affect-specific modulation of the N1m to shock-conditioned tones: magnetoencephalographic correlates. Eur. J. Neurosci. 37, 303–315. 10.1111/ejn.12043 23167712

[B15] Bröckelmann A.-K. Steinberg C. Elling L. Zwanzger P. Pantev C. Junghöfer M. (2011). Emotion-associated tones attract enhanced attention at early auditory processing: magnetoencephalographic correlates. J. Neurosci. 31, 7801–7810. 10.1523/JNEUROSCI.6236-10.2011 21613493 PMC6633129

[B16] Brugha T. Bebbington P. Tennant C. Hurry J. (1985). The list of threatening experiences: a subset of 12 life event categories with considerable long-term contextual threat. Psychol. Med. 15, 189–194. 10.1017/s003329170002105x 3991833

[B17] Carter R. M. O’Doherty J. P. Seymour B. Koch C. Dolan R. J. (2006). Contingency awareness in human aversive conditioning involves the middle frontal gyrus. Neuroimage 29, 1007–1012. 10.1016/j.neuroimage.2005.09.011 16246595

[B18] Caulfield M. D. McAuley J. D. Servatius R. J. (2013). Facilitated acquisition of eyeblink conditioning in those vulnerable to anxiety disorders. Front. Hum. Neurosci. 7:348. 10.3389/fnhum.2013.00348 23847516 PMC3701872

[B19] Chan C. K. Lovibond P. F. (1996). Expectancy bias in trait anxiety. J. Abnorm. Psychol. 105, 637–647. 10.1037/0021-843x.105.4.637 8952197

[B20] Corbetta M. Shulman G. L. (2002). Control of goal-directed and stimulus-driven attention in the brain. Nat. Rev. Neurosci. 3, 201–215. 10.1038/nrn755 11994752

[B21] Cosand L. D. Cavanagh T. M. Brown A. A. Courtney C. G. Rissling A. J. Schell A. M.. (2008). Arousal, working memory and conscious awareness in contingency learning. Conscious. Cogn. 17, 1105–1113. 10.1016/j.concog.2008.04.007 18573667

[B22] Davis M. (1998). Are different parts of the extended amygdala involved in fear versus anxiety? Biol. Psychiatry 44, 1239–1247. 10.1016/s0006-3223(98)00288-1 9861467

[B23] Davis M. Falls W. A. Gewirtz J. (2000). “Neural systems involved in fear inhibition: extinction and conditioned inhibition,” in Contemporary Issues in Modeling Psychopathology, eds Myslobodsky M. S. Weiner I. (US: Springer), 113–142.

[B24] Davis M. Walker D. L. Lee Y. (1997). Roles of the amygdala and bed nucleus of the stria terminalis in fear and anxiety measured with the acoustic startle reflex. Possible relevance to PTSD. Ann. N Y Acad. Sci. 821, 305–331. 10.1111/j.1749-6632.1997.tb48289.x 9238214

[B25] Deacon B. J. Abramowitz J. S. Woods C. M. Tolin D. F. (2003). The anxiety sensitivity index—revised: psychometric properties and factor structure in two nonclinical samples. Behav. Res. Ther. 41, 1427–1449. 10.1016/s0005-7967(03)00065-2 14583412

[B26] Eden A. S. Zwitserlood P. Keuper K. Junghöfer M. Laeger I. Zwanzger P.. (2014). All in its proper time: monitoring the emergence of a memory bias for novel, arousing-negative words in individuals with high and low trait anxiety. PLoS One 9:e98339. 10.1371/journal.pone.0098339 24887093 PMC4041778

[B27] Ehlers A. (1986). Angst-Sensitivitäts-Index (ASI). Göttingen: Unveröffentlichtes Manuskript, Georg-August-Universität.

[B28] Eysenck M. W. Derakshan N. Santos R. Calvo M. G. (2007). Anxiety and cognitive performance: attentional control theory. Emotion 7, 336–353. 10.1037/1528-3542.7.2.336 17516812

[B29] Fehm L. (2003). “Angstsensitivitätsindex (ASI),” in Angstdiagnostik, eds Hoyer J. Margraf J. (Berlin: Springer), 109–112.

[B30] Friedman B. H. (2007). An autonomic flexibility-neurovisceral integration model of anxiety and cardiac vagal tone. Biol. Psychol. 74, 185–199. 10.1016/j.biopsycho.2005.08.009 17069959

[B31] Gazendam F. J. Kamphuis J. H. Kindt M. (2013). Deficient safety learning characterizes high trait anxious individuals. Biol. Psychol. 92, 342–352. 10.1016/j.biopsycho.2012.11.006 23174693

[B32] Gershuny B. S. Sher K. J. (1998). The relation between personality and anxiety: findings from a 3-year prospective study. J. Abnorm. Psychol. 107, 252–262. 10.1037/0021-843x.107.2.252 9604554

[B33] Goodwin R. D. Faravelli C. Rosi S. Cosci F. Truglia E. de Graaf R.. (2005). The epidemiology of panic disorder and agoraphobia in Europe. Eur. Neuropsychopharmacol. 15, 435–443. 10.1016/j.euroneuro.2005.04.006 15925492

[B34] Gray J. R. Braver T. S. Raichle M. E. (2002). Integration of emotion and cognition in the lateral prefrontal cortex. Proc. Natl. Acad. Sci. U S A 99, 4115–4120. 10.1073/pnas.062381899 11904454 PMC122657

[B35] Green D. M. Swets J. A. (1966). Signal Detection Theory and Psychophysics. New York: Wiley.

[B36] Grillon C. (2002). Startle reactivity and anxiety disorders: aversive condtioning, context and neurobiology. Biol. Psychiatry 52, 958–975. 10.1016/s0006-3223(02)01665-7 12437937

[B37] Grillon C. Morgan C. A. I. (1999). Fear-potentiated startle conditioning to explicit and contextual cues in Gulf War veterans with posttraumatic stress disorder. J. Abnorm. Psychol. 108, 134–142. 10.1037/0021-843x.108.1.134 10066999

[B38] Guimarães F. S. Hellewell J. Hensman R. Wang M. Deakin J. F. W. (1991). Characterization of a psychophysiological model of classical fear conditioning in healthy volunteers: influence of gender, instruction, personality and placebo. Psychopharmacology (Berl) 104, 231–236. 10.1007/bf02244184 1876667

[B39] Hämäläinen M. S. Ilmoniemi R. J. (1994). Interpreting magnetic fields of the brain: minimum norm estimates. Med. Biol. Eng. Comput. 32, 35–42. 10.1007/bf02512476 8182960

[B40] Hauk O. (2004). Keep it simple: a case for using classical minimum norm estimation in the analysis of EEG and MEG data. Neuroimage 21, 1612–1621. 10.1016/j.neuroimage.2003.12.018 15050585

[B41] Hermann C. Ziegler S. Birbaumer N. Flor H. (2002). Psychophysiological and subjective indicators of aversive pavlovian conditioning in generalized social phobia. Biol. Psychiatry 52, 328–337. 10.1016/s0006-3223(02)01385-9 12208640

[B42] Heslegrave R. J. Ogilvie J. C. Furedy J. J. (1979). Measuring baseline-treatment differences in heart rate variability: variance versus successive difference mean square and beats per minute versus interbeat intervals. Psychophysiology 16, 151–157. 10.1111/j.1469-8986.1979.tb01461.x 424498

[B43] Holloway J. L. Trivedi P. Myers C. E. Servatius R. J. (2012). Enhanced conditioned eyeblink response acquisition and proactive interference in anxiety vulnerable individuals. Front. Behav. Neurosci. 6:76. 10.3389/fnbeh.2012.00076 23162449 PMC3499707

[B44] Holmes A. Nielsen M. K. Green S. (2008). Effects of anxiety on the processing of fearful and happy faces: an event-related potential study. Biol. Psychol. 77, 159–173. 10.1016/j.biopsycho.2007.10.003 18022310

[B45] Inslicht S. S. Metzler T. J. Garcia N. M. Pineles S. L. Milad M. R. Orr S. P.. (2013). Sex differences in fear conditioning in posttraumatic stress disorder. J. Psychiatr. Res. 47, 64–71. 10.1016/j.jpsychires.2012.08.027 23107307 PMC3806498

[B46] Jackson E. D. Payne J. D. Nadel L. Jacobs W. J. (2006). Stress differentially modulates fear conditioning in healthy men and women. Biol. Psychiatry 59, 516–522. 10.1016/j.biopsych.2005.08.002 16213468

[B47] Joos E. Vansteenwegen D. Hermans D. (2012). Worry as a predictor of fear acquisition in a nonclinical sample. Behav. Modif. 36, 723–750. 10.1177/0145445512446477 22679241

[B48] Jorm A. F. Christensen H. Henderson A. S. Jacomb P. A. Korten A. E. Rodgers B. (2000). Predicting anxiety and depression from personality: is there a synergistic effect of neuroticism and extraversion? J. Abnorm. Psychol. 109, 145–149. 10.1037/0021-843x.109.1.145 10740946

[B49] Junghöfer M. Elbert T. Tucker D. M. Rockstroh B. (2000). Statistical control of artifacts in dense array EEG/MEG studies. Psychophysiology 37, 523–532. 10.1111/1469-8986.3740523 10934911

[B50] Klinkenberg I. Bröckelmann A. Dobel C. Kirschbaum C. Plessow F. Junghöfer M. (2011). “Scent of a man–pheromone-enhanced processing of male faces,” in Poster Session Presented at the XI International Conference on Cognitive Neuroscience (ICON XI) (Palma de Mallorca, Spain).

[B51] Laux L. Glanzmann P. Schaffner P. Spielberger C. D. (1981). Das State-Trait-Angstinventar. Theoretische Grundlagen und Handanweisung [The State-Trait-Anxiety Inventory. Theoretical Basics and Instructions]. Weinheim: Beltz Test.

[B52] Lecrubier Y. Sheehan D. V. Weiller E. Amorim P. Bonora I. Harnett Sheehan K. (1997). The Mini International Neuropsychiatric Interview (MINI). A short diagnostic structured interview: reliability and validity according to the CIDI. Eur. Psychiatry 12, 224–231. 10.1016/s0924-9338(97)83296-8

[B53] Lissek S. Pine D. S. Grillon C. (2006). The strong situation: a potential impediment to studying the psychobiology and pharmacology of anxiety disorders. Biol. Psychol. 72, 265–270. 10.1016/j.biopsycho.2005.11.004 16343731

[B54] Lissek S. Powers A. S. McClure E. B. Phelps E. A. Woldehawariat G. Grillon C.. (2005). Classical fear conditioning in the anxiety disorders: a meta-analysis. Behav. Res. Ther. 43, 1391–1424. 10.1016/j.brat.2004.10.007 15885654

[B55] Lonsdorf T. B. Kalisch R. (2011). A review on experimental and clinical genetic associations studies on fear conditioning, extinction and cognitive-behavioral treatment. Transl. Psychiatry 1:e41. 10.1038/tp.2011.36 22832657 PMC3309482

[B56] Lundqvist D. Flykt A. Öhman A. (1998). The Karolinska Directed Emotional Faces–KDEF.CD ROM from Department of Clinical Neuroscience, Psychology Section. Karolinska Institutet.

[B57] MacLeod C. Mathews A. Tata P. (1986). Attentional bias in emotional disorders. J. Abnorm. Psychol. 95, 15–20. 10.1037/0021-843x.95.1.15 3700842

[B58] Maris E. Oostenveld R. (2007). Nonparametric statistical testing of EEG- and MEG-data. J. Neurosci. Methods 164, 177–190. 10.1016/j.jneumeth.2007.03.024 17517438

[B59] McNally R. J. Bryant R. A. Ehlers A. (2003). Does early psychological intervention promote recovery from posttraumatic stress? Psychol. Sci. Public Interes. 4, 45–79. 10.1111/1529-1006.01421 26151755

[B60] Milad M. R. Goldstein J. M. Orr S. P. Wedig M. M. Klibanski A. Pitman R. K.. (2006). Fear conditioning and extinction: influence of sex and menstrual cycle in healthy humans. Behav. Neurosci. 120, 1196–1203. 10.1037/0735-7044.120.5.1196 17201462

[B61] Moriya J. Sugiura Y. (2012). High visual working memory capacity in trait social anxiety. PLoS One 7:e34244. 10.1371/journal.pone.0034244 22496783 PMC3322141

[B62] Mosig C. Merz C. J. Mohr C. Adolph D. Wolf O. T. Schneider S.. (2014). Enhanced discriminative fear learning of phobia-irrelevant stimuli in spider-fearful individuals. Front. Behav. Neurosci. 8:328. 10.3389/fnbeh.2014.00328 25324745 PMC4181334

[B63] Motrico E. Moreno-Küstner B. de Dios L. J. Torres-Gonzáles F. King M. Nazareth I.. (2013). Psychometric properties of the list of threatening experiences-LTE and its association with psychosocial factors and mental disorders according to different scoring methods. J. Affect. Disord. 150, 931–940. 10.1016/j.jad.2013.05.017 23726778

[B64] Nelting M. Rienhoff N. K. Hesse G. Lamparter U. (2002). Die Erfassung des subjektiven Leidens unter Hyperakusis mit einem Selbstbeurteilungsbogen zur Geräuschüberempfindlichkeit (GÜF). [The assessment of subjective distress related to hyperacusis with a self-rating questionnaire on hypersensitivity to sound]. Laryngo Rhino Otology 81, 327–334. 10.1055/s-2002-28342 12001021

[B65] Orr S. P. Metzger L. J. Lasko N. B. Macklin M. L. Peri T. Pitman R. K. (2000). De novo conditioning in trauma-exposed individuals with and without posttraumatic stress disorder. J. Abnorm. Psychol. 109, 290–298. 10.1037/0021-843x.109.2.290 10895567

[B66] Otto M. W. Leyro T. M. Christian K. Deveney C. M. Resse H. Pollack M. H.. (2007). Prediction of “fear” in healthy control participants in a de novo fear-conditioning paradigm. Behav. Modif. 31, 32–51. 10.1177/0145445506295054 17179530 PMC1764631

[B67] Pape H.-C. Pare D. (2010). Plastic synaptic networks of the amygdala for the acquisition, expression and extinction of conditioned fear. Physiol. Rev. 90, 419–463. 10.1152/physrev.00037.2009 20393190 PMC2856122

[B68] Pastor M. C. Moltó J. Vila J. Lang P. J. (2003). Startle reflex modulation, affective ratings and autonomic reactivity in incarcerated Spanish psychopaths. Psychophysiology 40, 934–938. 10.1111/1469-8986.00111 14986846

[B69] Pavlov I. P. (1927). Conditioned Reflexes: An Investigation of the Physiological Activity of the Cerebral Cortex. London: Oxford University Press. 10.5214/ans.0972-7531.1017309 PMC4116985 25205891

[B70] Peri T. Ben-Shakhar G. Orr S. P. Shalev A. Y. (2000). Psychophysiologic assessment of aversive conditioning in posttraumatic stress disorder. Biol. Psychiatry 47, 512–519. 10.1016/s0006-3223(99)00144-4 10715357

[B71] Pessoa L. (2010). Emergent processes in cognitive-emotional interactions. Dialogues Clin. Neurosci. 12, 433–448. 21319489 10.31887/DCNS.2010.12.4/lpessoa PMC3117594

[B72] Peyk P. De Cesarei A. Junghöfer M. (2011). ElectroMagnetoEncephalography software: overview and integration with other EEG/MEG toolboxes. Comput. Intell. Neurosci. 2011:861705. 10.1155/2011/861705 21577273 PMC3090751

[B73] Phillips P. J. Moon H. Rizvi S. A. Rauss P. J. (2000). The FERET evaluation methodology for face-recognition algorithms. IEEE Trans. Pattern Anal. Mach. Intell. 22, 1090–1104. 10.1109/34.879790

[B74] Phillips P. J. Wechsler H. Huang J. Rauss P. J. (1998). The FERET database and evaluation procedure for face-recognition algorithms. Image Vis. Comput. 16, 295–306. 10.1016/s0262-8856(97)00070-x

[B75] Pitman R. K. Orr S. P. (1986). Test of the conditioning model of neurosis: differential aversive conditioning of angry and neutral facial expressions in anxiety disorder patients. J. Abnorm. Psychol. 95, 208–213. 10.1037/0021-843x.95.3.208 3745641

[B76] Pleyers G. Corneille O. Luminet O. Yzerbyt V. (2007). Aware and (dis)liking: item-based analyses reveal that valence acquisition via evaluative conditioning emerges only when there is contingency awareness. J. Exp. Psychol. Learn. Mem. Cogn. 33, 130–144. 10.1037/0278-7393.33.1.130 17201557

[B77] Raes A. K. De Raedt R. Verschuere B. De Houwer J. (2009). Failure to loose fear: the impact of cognitive load and trait anxiety on extinction. Behav. Res. Ther. 47, 1096–1101. 10.1016/j.brat.2009.08.002 19716551

[B78] Rehbein M. A. Steinberg C. Wessing I. Pastor M. C. Zwitserlood P. Keuper K.. (2014). Rapid plasticity in the prefrontal cortex during affective associative learning. PLoS One 9:e110720. 10.1371/journal.pone.0110720 25333631 PMC4204938

[B79] Reiss S. Peterson R. A. Gursky D. M. McNally R. J. (1986). Anxiety sensitivity, anxiety frequency and the prediction of fearfulness. Behav. Res. Ther. 24, 1–8. 10.1016/0005-7967(86)90143-9 3947307

[B80] Salemink E. van den Hout M. A. Kindt M. (2007). Selective attention and threat: quick orienting versus slow disengagement and two versions of the dot probe task. Behav. Res. Ther. 45, 607–615. 10.1016/j.brat.2006.04.004 16769035

[B81] Simon J. R. (1990). “The effects of an irrelevant directional cue on human information processing,” in Stimulus-Response Compatibility: An Integrated Perspective, eds Proctor R. W. Reeve T. G. (Amsterdam: Elsevier Science Publishers B.V.), 31–86.

[B82] Simon J. R. Rudell A. P. (1967). Auditory S-R compatibility: the effect of an irrelevant cue on information processing. J. Appl. Psychol. 51, 300–304. 10.1037/h0020586 6045637

[B83] Somerville L. H. Whalen P. J. Kelley W. M. (2010). Human bed nucleus of the stria terminalis indexes hypervigilant threat monitoring. Biol. Psychiatry 68, 416–424. 10.1016/j.biopsych.2010.04.002 20497902 PMC2921460

[B84] Steinberg C. Bröckelmann A.-K. Rehbein M. Dobel C. Junghöfer M. (2013). Rapid and highly resolving associative affective learning: convergent electro- and magnetoencephalographic evidence from vision and audition. Biol. Psychol. 92, 526–540. 10.1016/j.biopsycho.2012.02.009 23481617

[B85] Steinberg C. Dobel C. Schupp H. T. Kissler J. Elling L. Pantev C.. (2012). Rapid and highly resolving: affective evaluation of olfactorily conditioned faces. J. Cogn. Neurosci. 24, 17–27. 10.1162/jocn_a_00067 21671742

[B86] Stevens S. Rist F. Gerlach A. L. (2009). Influence of alcohol on the processing of emotional facial expressions in individuals with social phobia. Br. J. Clin. Psychol. 48, 125–140. 10.1348/014466508x368856 18851774

[B87] Stevens S. Rist F. Gerlach A. L. (2011). Eye movement assessment in individuals with social phobia: differential usefulness for varying presentation times? J. Behav. Ther. Exp. Psychiatry 42, 219–224. 10.1016/j.jbtep.2010.11.001 21315885

[B88] Taylor S. Cox B. J. (1998). An expanded anxiety sensitivity index: evidence for a hierarchie structure in a clinical sample. J. Anxiety Disord. 12, 463–483. 10.1016/S0887-6185(98)00028-0 9801964

[B89] Tempesta D. Couyoumdjian A. Curcio G. Moroni F. Marzano C. De Gennaro L.. (2010). Lack of sleep affects the evaluation of emotional stimuli. Brain Res. Bull. 82, 104–108. 10.1016/j.brainresbull.2010.01.014 20117179

[B90] Torrents-Rodas D. Fullana M. A. Bonillo A. Caseras X. Andión O. Torrubia R. (2013). No effect of trait anxiety on differential fear conditioning or fear generalization. Biol. Psychol. 92, 185–190. 10.1016/j.biopsycho.2012.10.006 23131617

[B91] Tottenham N. Tanaka J. W. Leon A. C. McCarry T. Nurse M. Hare T. A.. (2009). The NimStim set of facial expressions: judgments from untrained research participants. Psychiatry Res. 168, 242–249. 10.1016/j.psychres.2008.05.006 19564050 PMC3474329

[B92] Ungerleider L. G. Courtney S. M. Haxby J. V. (1998). A neural system for human visual working memory. Proc. Natl. Acad. Sci. U S A 95, 883–890. 10.1073/pnas.95.3.883 9448255 PMC33812

[B93] Vaidyanathan U. Patrick C. J. Cuthbert B. N. (2009). Linking dimensional models of internalizing psychopathology to neurobiological systems: affect-modulated startle as an indicator of fear and distress disorders and affiliated traits. Psychol. Bull. 135, 909–942. 10.1037/a0017222 19883142 PMC2776729

[B94] Wickens T. D. (2002). Elementary Signal Detection Theory. New York: Oxford University Press.

[B95] Wilhelm F. H. Peyk P. (2005). ANSLAB 4.0: autonomic nervous system laboratory [software]. Available online at: http://www.sprweb.org

[B96] Wittchen H.-U. Jacobi F. (2005). Size and burden of mental disorders in Europe–a critical review and appraisal of 27 studies. Eur. Neuropsychopharmacol. 15, 357–376. 10.1016/j.euroneuro.2005.04.012 15961293

[B97] Zorawski M. Cook C. A. Kuhn C. M. LaBar K. S. (2005). Sex, stress and fear: individual differences in conditioned learning. Cogn. Affect. Behav. Neurosci. 5, 191–201. 10.3758/cabn.5.2.191 16180625

